# Analyzing latent state-trait and multiple-indicator latent growth curve models as multilevel structural equation models

**DOI:** 10.3389/fpsyg.2013.00975

**Published:** 2013-12-30

**Authors:** Christian Geiser, Jacob Bishop, Ginger Lockhart, Saul Shiffman, Jerry L. Grenard

**Affiliations:** ^1^Department of Psychology, Utah State UniversityLogan, UT, USA; ^2^Department of Psychology, University of PittsburghPittsburgh, PA, USA; ^3^School of Community and Global Health, Claremont Graduate UniversityClaremont, CA, USA

**Keywords:** latent state-trait analysis, multiple-indicator latent growth curve models, multilevel structural equation models, individually-varying and unequally-spaced time points, mixed-effects models, ecological momentary assessment data, intensive longitudinal data

## Abstract

Latent state-trait (LST) and latent growth curve (LGC) models are frequently used in the analysis of longitudinal data. Although it is well-known that standard single-indicator LGC models can be analyzed within either the structural equation modeling (SEM) or multilevel (ML; hierarchical linear modeling) frameworks, few researchers realize that LST and multivariate LGC models, which use multiple indicators at each time point, can also be specified as ML models. In the present paper, we demonstrate that using the ML-SEM rather than the SL-SEM framework to estimate the parameters of these models can be practical when the study involves (1) a large number of time points, (2) individually-varying times of observation, (3) unequally spaced time intervals, and/or (4) incomplete data. Despite the practical advantages of the ML-SEM approach under these circumstances, there are also some limitations that researchers should consider. We present an application to an ecological momentary assessment study (*N* = 158 youths with an average of 23.49 observations of positive mood per person) using the software Mplus (Muthén and Muthén, 1998–2012) and discuss advantages and disadvantages of using the ML-SEM approach to estimate the parameters of LST and multiple-indicator LGC models.

Many researchers in psychology are interested in modeling the longitudinal dynamics of constructs such as extraversion, cognitive abilities, subjective well-being, or mood states. Popular latent variable models for the analysis of longitudinal data include (1) models for measuring short-term state-variability processes around a fixed trait level and (2) models for measuring long-lasting trait changes (Eid, 2007). Latent state-trait (LST) models (Steyer et al., 1992, 1999, 2012) can be seen as prototypical models for measuring short-term state-variability processes that do not involve actual trait changes (Geiser et al., submitted). LST analyses are often used to determine the degree of across-time consistency vs. situation-specificity of individual differences. LST models are suitable when the underlying longitudinal process is characterized by a stable trait with short-term and reversible situation-specific deviations from the general trait level. Examples of such state-fluctuation processes include psychological constructs like mood states, which are characterized by a relatively stable trait level, but situation-specific deviations (ups and downs) from this general level (e.g., Eid et al., 1999). LST models typically use multiple indicators of the attribute under study, although single-indicator models have been presented in the literature as well (Kenny and Zautra, 1995; for a review of single-indicator LST models see Cole et al., 2005).

In contrast to LST models, latent growth curve (LGC) models (McArdle and Epstein, 1987; Meredith and Tisak, 1990; Bollen and Curran, 2006; Duncan et al., 2006) are designed to capture long-lasting and potentially irreversible trait changes across time (Geiser et al., submitted, 2013). Examples of trait-change processes include changes in height from early childhood to adolescence or lasting changes in psychological functioning after psychological interventions such as psychotherapy. LGC models often assume that the trait-change process follows a specific functional form (e.g., linear, quadratic, or cubic) that is the same for all individuals in the population [Note that so-called growth mixture models (e.g., Muthén, 2004) relax this assumption and allow for different trajectories in previously unknown subpopulations]. LGC models are thus suitable when the longitudinal process cannot be characterized by mere fluctuations around a stable trait, but when individuals' trait values themselves change over time.

In contrast to the majority of LST applications, most applications of LGC models to date have used a single repeatedly measured observed variable as indicator of the latent growth process. On the other hand, there is consensus among methodological researchers that the use of multiple indicators at each measurement occasion can help overcome important limitations of single-indicator LGC models. First, single-indicator LGC models make the implicit assumption of measurement equivalence (equal factor loading and intercept parameters across time for identical measured variables; e.g., Sayer and Cumsille, 2001; for general introductions to the issue of measurement equivalence, see Meredith, 1993 or Millsap, 2011), but this assumption is not testable in single-indicator models. Multiple-indicator LGC models allow testing this assumption (Ferrer et al., 2008).

Second, single-indicator LGC models imply that the construct under study is perfectly trait-like and does not depend on situational influences (Geiser et al., 2013). If situational effects are present, these will be confounded with measurement error in single-indicator models, leading to underestimated reliabilities in actual applications. Multiple-indicator LGC models avoid this problem, because they allow separating reliable occasion-specific (state residual) variance from reliable growth variance and random measurement error variance.

Third, single-indicator models have been shown to provide less statistical power for detecting individual differences in change over time compared to multiple-indicator LGC models (von Oerzen et al., 2010). Fourth, given that single-indicator LGC models use just one indicator of the construct, potential method-specific variance of this indicator (e.g., self-report bias) cannot be examined. Multiple-indicator extension of LGC models can in part cure this problem as well.

The most prominent multiple-indicator LGC model to date is the *second-order* or *curve-of-factors model* proposed by McArdle (1988; see also Hancock et al., 2001; Leite, 2007). Two related approaches that relax some of the rather restrictive assumptions of the second-order LGC model have recently been presented by Eid et al. (2012) as well as Bishop et al. (submitted). In the present paper, we focus on these less restrictive approaches. Their relationship to McArdle's (1988) second-order LGC is discussed in detail in Bishop et al. (submitted).

## Single- vs. multi-level specification of LST and LGC models

Both LST and LGC models are commonly applied in the conventional single-level (SL) structural equation modeling (SEM) framework. In addition, many researchers apply single-indicator LGC models within the hierarchical linear modeling or multilevel (ML) analysis framework (e.g., Bryk and Raudenbush, 1987; Mehta and West, 2000). This is possible, because longitudinal (or repeated measures) data has a hierarchical or ML structure as we explain in detail below. Although this is a well-known fact for single-indicator LGC models, we know of no application of LST or multiple-indicator LGC models that have specified these models as ML models. In the present article, we show how both LST and multiple-indicator LGC models can be specified as ML models and discuss advantages and limitations of estimating the parameters of these models in the ML as compared to the SL-SEM framework.

## Longitudinal data as ML data

It has long been recognized that longitudinal (or repeated-measures) data, in which the same individuals are assessed at multiple time points, have a hierarchical (or ML) structure (e.g., Bryk and Raudenbush, 1987; Luke, 2004 for general introductions to ML modeling techniques, see Kreft and de Leeuw, 1998; Hox, 2002; or Snijders and Bosker, 1999; Luke, 2004). That is, in longitudinal data, the repeated observations or measurements (Level 1) are nested within individuals (Level 2)^1^. As a consequence, longitudinal data can be analyzed with ML modeling techniques.

Estimating the parameters of longitudinal models (such as LST and LGC models) based on long-format data using ML analysis techniques rather than wide-format data and SL statistical procedures can have practical advantages under certain conditions (Plewis, 2005). First, when the number of time points is large, the ML framework results in a considerably more “compact” specification of the models compared to the corresponding SL-SEM approach, in which a separate measurement model has to be set up for each time point. Second, when the study involves individually-varying times of observations, the proper specification of a growth model within the SL-SEM framework can be quite laborious, because the growth factor loadings have to be adjusted for individual differences in times of observations. The situation becomes even more complicated when, third, time points do not only vary between individuals, but are also unequally-spaced within individuals. In this case, the specification of growth models within the SL-SEM framework can be a lot more complex than the corresponding specification as a ML model and sometimes virtually infeasible.

Fourth, longitudinal studies are typically prone to missing data due to occasional non-response or permanent drop-out of the study. As long as missing data fulfills the *missing at random* (MAR) or *missing completely at random* (MCAR) assumptions, modern missing data handling methods such as full information maximum likelihood (FIML) or multiple imputation (MI) can be used to take all available data into account (see Enders, 2010, for details). Although FIML or MI can be used to properly account for MAR and MCAR missing data in the SL-SEM framework, the multilevel approach handles these situations in a more straightforward way (Strategies for analyzing LGC models with *missing not at random* [MNAR] data were recently discussed by Enders, 2011).

The four above-mentioned conditions (a large number of individually-varying and unequally-spaced time points with missing data) are frequently encountered in psychological research. As an example, consider ecological momentary assessment (EMA) studies (sometimes also referred to as *ambulatory assessment studies*; Shiffman et al., 2008), in which individuals are measured on multiple time points each day for one or more weeks, resulting in a large number of measurements per individual. EMA methods often sample subjects' states at random times, or at times associated with particular, asynchronous events, such as episodes of eating. In these studies, observations therefore typically do not take place at the same time point for all individuals and also typically are not equally spaced in time. In these cases, the specification of longitudinal models remains straightforward in the ML framework, whereas it can become quite complicated within the SL-SEM approach.

In the ML approach, the relatively simple inclusion of a time variable solves the problem of individually varying and unequally-spaced time points. Another potential advantage of the ML framework is that missing values due to drop-out over time are easily handled in these models, because the ML approach does not require all individuals to have the same number of time points. As a consequence, no imputation or other missing data algorithms need to be explicitly selected; the missing data problem is handled “implicitly” by FIML estimation.

The fact that conventional single-indicator LGC models for a single measured variable at each time point can be more efficiently specified within the ML framework is well-known among many researchers. Less well-appreciated is the fact that longitudinal SEM models that use multiple indicators per time point (e.g., standard LST and multiple-indicator LGC models) can also be specified within the ML framework. The reason may be that multiple-indicator models require the use of *multivariate* ML models, that is, of ML-SEM techniques, which are relatively new (for general introductions to ML-SEM, see Mehta and Neale, 2005 or Muthén, 1994).

In the following sections, we first review the formulations of both LST and multiple-indicator LGC models as conventional SL-SEM models. Using path diagrams, we then show how for each type of model, an equivalent ML-SEM formulation can be obtained. Subsequently, we present ML-SEM applications of both LST and LGC models to actual data and discuss advantages and limitations of the ML-SEM approach.

## LST theory and models

LST analyses are based on multiple repeatedly-measured observed variables *Y*_*it*_ (*i* = indicator, *i* = 1, …, *j*, …, *m*; *t* = time point, *t* = 0,…, *s*,…, *n*) of the same construct (e.g., anxiety, subjective well-being, extraversion etc.). Indicators for a construct in an LST model could, for example, be different items, scale scores, or physiological measures. LST theory assumes that each observed variable is a function of an indicator-specific latent trait variable ξ_*it*_ that characterizes person-specific (trait) effects, an indicator-specific latent state residual variable ζ_*it*_ that characterizes effects of the situation or person × situation interactions for that indicator, and a residual variable ε_*it*_ that reflects random measurement error (for explicit mathematical definitions and a detailed discussion of the properties of the latent variables in LST theory see, e.g., Steyer et al., 1992, 2012; or Geiser and Lockhart, 2012):

(1) $$Y_{it}=\xi_{it}+\zeta_{it}+\varepsilon_{it}.$$

By definition in LST theory, the latent state residuals and error variables have zero means, that is, *E*(ζ_*it*_) = *E* (ε_*it*_) = 0. Furthermore, the latent trait variables ξ_*it*_ are by definition uncorrelated with all latent state residual variables ζ_*it*_ and with all error variables ε_*it*_. In the present paper, we further assume that all ζ_*it*_ are uncorrelated with each other and with all ε_*it*_, and that there are no correlations among the error variables ε_*it*_ (Note that models could be specified in which some of these assumptions are relaxed; such models were discussed, for example, by Cole et al., 2005).

### The singletrait-multistate (STMS) model

A simple and commonly used LST model that can be formulated based on the decomposition in Equation 1 is the so-called singletrait-multistate (STMS) model (e.g., Geiser et al., submitted). In this model, it is assumed that all latent trait variables are congeneric, that is, that they are linear functions of a common latent trait factor ξ:

(2) $$\xi_{it}=\alpha_{i}+\lambda_{i}\xi .$$

Furthermore, it is assumed that all variables measured at the same time point share the same occasion-specific (state-variability) process within scaling differences:

(3) $$\zeta_{it}=\gamma_{i}\zeta_{t}.$$

Note that time-invariance of the constant scaling parameters (intercepts α_*i*_ and loadings λ_*i*_ and γ_*i*_) is assumed in this model. This is indicated by the fact that the index for the time point (*t*) has been omitted from these parameters. Time-invariance of factor loadings and intercepts is required in the LST model if the model is to be interpreted as a pure state-variability model. If specified with non-invariant parameters, the model could capture true trait changes and could thus not be interpreted as a pure state-variability model (For more details on the role of measurement [non]equivalence in LST models, see Geiser et al., submitted).

In summary, each observed variable *Y*_*it*_ in the STMS model is a function of a constant time-invariant intercept parameter α_*i*_, a common latent trait factor ξ that is shared by all observed variables, a common latent state residual variable ζ_*t*_ that is shared by all observed variables that are measured at the same time point, and a variable-specific measurement error variable ε_*it*_:

(4) $$Y_{it}=\alpha_{i}+\lambda_{i}\xi +\gamma_{i}\zeta_{t}+\varepsilon_{it}.$$

The variable-specific factor loading parameters λ_*i*_ and γ_*i*_ are included to allow for potential differences between indicators in terms of the units of measurement or test discrimination (in the sense of item response theory). The variable-specific intercepts α_*i*_ are included to allow for differences in the origin of measurement.

**Figure 1:**
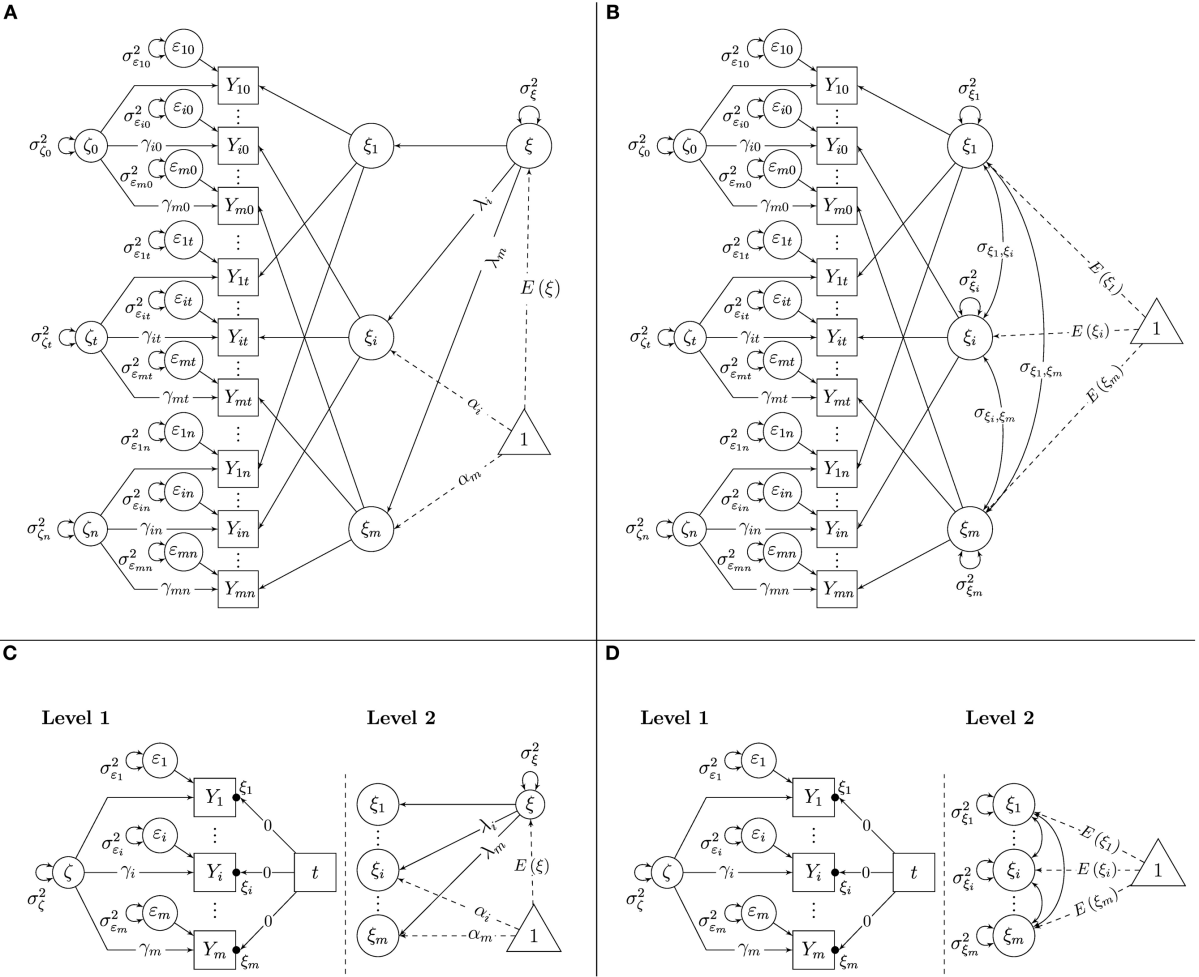
**Latent state-trait models. (A)** STMS model as SL-SEM model. **(B)** MTMS model as SL-SEM model. **(C)** STMS model as ML-SEM model. **(D)** MTMS model as ML-SEM model.

The model can be identified by setting the factor loading parameters of one reference indicator (e.g., *Y*_1*t*_) to 1 (i.e., λ_1_ = γ_1_ = 1) and the constant intercepts of the same indicator to 0 (i.e., α_1_ = 0) at all time points. Then, the remaining intercepts, loadings, the variances of all latent variables, and the mean of the latent trait factor, *E*(ξ), are identified and can be estimated as free parameters as long as *m* ≥ 3 and *n* ≥ 2 (For *m* = 2, the model can be identified by additionally setting all γ_*i*_ = 1 for all *i* or by allowing each latent state residual factor to be correlated with at least one external variable in the model). Figure 1A shows a path diagram of the SL-SEM parameterization of the STMS model with the above-mentioned identifying constraints on loadings and intercepts^2^.

### The multitrait-multistate (MTMS) model

The STMS model may be too restrictive for many practical applications, because it requires all observed variables to share exactly the same trait factor (within scaling differences). This assumption is violated when there are method-specific effects or when observed variables measure slightly different traits or distinct facets of a construct. A useful alternative model for less than perfectly unidimensional indicators is the multitrait-multistate (MTMS) model that allows for variable-specific latent trait variables ξ _*i*_ (e.g., Eid et al., 1999; Geiser and Lockhart, 2012). In the MTMS model, it is assumed that the trait variables are identical for the same indicator *i*:

(5) $$\xi_{it}=\xi_{it^{\prime }}=\xi_{i}.$$

As a result, each indicator has its own trait factor ξ_*i*_ in the MTMS model. In addition, we make the same assumption of occasion-specific congenerity of latent state residual variables as in the STMS model (see Equation 3). The complete measurement equation for the MTMS model is given by:

(6) $$Y_{it}=\xi_{i}+\gamma_{i}\zeta_{t}+\varepsilon_{it}.$$

The indicator-specific traits ξ_*i*_ can be correlated with each other. Correlations close to 1 indicate that the trait components of different indicators are essentially homogeneous, whereas low to moderate correlations indicate that different indicators measure different traits, different facets of a construct, or that the indicators show high method-specificity. The MTMS model can be identified by setting the latent state residual factor loading parameters of one reference indicator (e.g., *Y*_1*t*_) to 1 (i.e., γ_1_ = 1). Furthermore, all trait factor loadings in the MTMS model have to be set to 1 (there is an implicit coefficient of 1 before ξ_*i*_ in Equation 6) and all constant intercept parameters have to be set to zero (there is no additive constant in Equation 6). Then, the remaining state residual factor loadings, the variances of all latent variables, the means of the latent trait factors, *E*(ξ_*i*_), and their covariances are identified and can be estimated as free parameters as long as *m* ≥ 3 and *n* ≥ 2 (For *m* = 2, the model can be identified by additionally setting γ_*i*_ = 1 for all *i* or by allowing each latent state residual factor to be correlated with at least one external variable in the model). Figure 1B shows a path diagram of the SL-SEM parameterization of the MTMS model with the above-mentioned identifying constraints on loadings and intercepts.

### The STMS and MTMS models as multilevel models

In the ML-SEM specification of the STMS and MTMS models, effects of the situation and/or person × situation interactions as well as the effects of random measurement error are modeled on Level 1, because Level 1 represents the measurements at the different time points that are nested within the individuals (Level-2 units). The effects of the persons (i.e., the trait effects) are captured on Level 2. Technically, the trait effects are treated as random intercept parameters in (multivariate) linear regressions of the indicators on time (*t*). These regressions on *t* are estimated at Level 1. The random intercepts from these regressions are the latent trait variables. The means, variances, and covariances of the latent trait variables are estimated on Level 2.

Figure 1C shows a path diagram of the ML-SEM parameterization of the STMS model. In our ML-SEM path diagrams, we adopt the conventions for depicting Level-1 and Level-2 parameters in ML models proposed by Muthén and Muthén (1998–2012). According to these conventions, random intercept parameters are indicated with a dot (•) between the arrow pointing from an independent variable (in this case the time variable *t*) to an outcome variable (in this case the observed indicator variables *Y*_*i*_) and the box representing that outcome variable (see Figures 1C,D). In our figures, we label the random intercepts with the appropriate latent variable name (here ξ_*i*_).

The left panel of Figure 1C shows the Level-1 part of the model. On Level 1, three different effects are modeled: (1) the effect of measurement error as represented by the error variance parameters *Var*(ε_*i*_), the effect of the common latent state residual factor that is shared between indicators within scaling differences as represented by the variance parameter *Var*(ζ) and the factor loadings γ_*i*_, and (3) the effect of time (*t* = 0,…, *s*,…, *n*), as represented by the random intercepts in the regression of the indicators on the manifest time variable *t*.

Note that the slope coefficients in the regression on *t* are fixed at zero for all indicators. It may seem odd that the observed indicator variables *Y*_*i*_ are regressed on time with a regression slope coefficient fixed to zero in this model. However, we chose this way of representing the model for two reasons: this parameterization (1) helps clarifying the connection between LST and LGC models and (2) allows implicit restrictions to be tested in the LST model. The slope coefficients have to be fixed to zero in this model, because in LST models, the trait values are not supposed to change across time (Geiser et al., submitted). Hence, there is no effect of time on the indicators in LST models. This assumption can be tested in the model by comparing the fit of this model to the fit of a model in which the coefficients are freely estimated as fixed or random slopes (random slope models are presented later).

Even though the slope coefficients in the regressions on time are set to zero for all indicators, these regressions include a non-zero random intercept parameter for each outcome variable. These random intercepts are the indicator-specific latent trait variables ξ_*i*_. Conceptually, these trait variables are random intercepts in the ML-SEM specification, because the trait scores can vary between individuals (i.e., across Level-2 units). In the STMS model, it is assumed that all random intercepts are linear functions of a common random intercept (i.e., a common trait factor ξ). This relationship between the indicator-specific random intercepts ξ_*i*_ and the common trait ξ is modeled on Level 2 in the ML-SEM specification (The right panel of Figure 1C shows that the random intercepts ξ_*i*_ are functions of the common latent trait variable ξ). Therefore, on Level 2, the STMS model only estimates a single latent trait mean *E*(ξ) and variance parameter *Var*(ξ) as well as intercepts α_*i*_ and factor loadings λ_*i*_ as constant scaling parameters.

An interesting aspect of the ML formulation of this model is the fact that time-invariance of the fixed intercepts (α_*i*_ parameters) and loadings (λ_*i*_ and γ_*i*_ parameters) is implicitly assumed or “built-in” (which is not the case in the SL-SEM formulation of this model). This measurement equivalence assumption makes sense and is required in LST models if these models are to be interpreted as pure state-variability models (Geiser et al., submitted). Furthermore, the ML-SEM formulation of the STMS model implicitly constrains the Level-1 variance parameters [*Var*(ζ) and *Var*(ε_*i*_)] to be time-invariant. These constraints are not necessary for the proper interpretation of the model, and they may be too restrictive in specific empirical applications as discussed later. On the other hand, they contribute to model parsimony.

Figure 1D shows the ML-SEM version of the MTMS model in a path diagram. It can be seen that the only difference between Figure 1C (STMS) and Figure 1D (MTMS) is that the random intercepts in Figure 1D are not assumed to be functions of a common trait ξ on Level 2. Instead, each indicator-specific trait factor ξ_*i*_ has its own mean and variance parameter in the MTMS model. Furthermore, all indicator-specific traits can be correlated (covariance parameters on Level 2). The Level-1 model is the same as in the STMS approach and captures situation-specific effects and random measurement error with time-invariant loadings and variance parameters.

### Variance components and coefficients in the STMS and MTMS models

Both the STMS and the MTMS model allow for the definition of variance components due to person-specific (trait) effects, situational and/or person × situation interaction effects, and random measurement error. Based on the variance decomposition, coefficients can be defined that capture consistency (*Con*), occasion-specificity (*OSpec*), and reliability (*Rel*; Steyer et al., 1992). Table 1 contains the corresponding equations for all ML-SEM models presented in this paper (including the LGC models discussed below). The *Con* coefficient gives the proportion of variance that is due to person-specific (i.e., trait and/or trait-change) effects. The *OSpec* coefficient gives the proportion of variance that is due to situational and/or person × situation interaction effects. Note that *Con* and *OSpec* can be defined for either the observed variables *Y*_*it*_ (that typically contain measurement error) or the underlying true score variables τ_*it*_ (which are by definition free of measurement error). In case of the observed variables, the sum of *Con*(*Y_it_*) + *OSpec*(*Y*_*it*_) yields the total amount of reliable score variance, that is, the *Rel* coefficient. In the case of the true scores, the sum *Con*(τ_*it*_) + *OSpec*(τ_*it*_) equals 1. *Con*(τ_*it*_) and *OSpec*(τ_*it*_) make it easier to determine what percentage of the true score (i.e., reliable) variance is stable vs. situation-dependent.

**Table 1:** **Variance decomposition and coefficients in the ML-SEM versions of the MTMS, STMS, GSG, and ISG Models**.

**Description**	**Equation**
**STMS**	
Variance decomposition	$var{\left(Y\right)}_{i}=\lambda_{i}^{2}\;var\left(\xi \right)+\gamma_{i}^{2}\;var\left(\zeta \right)+var\left(\varepsilon_{i}\right)$
Indicator consistency	$Con\left(Y_{i}\right)=\frac{\lambda_{i}^{2}\;var\left(\xi \right)}{\lambda_{i}^{2}\;var\left(\xi \right)+\gamma_{i}^{2}\;var\left(\zeta \right)+var\left(\varepsilon_{i}\right)}$
Indicator occasion-specificity	$OSpec\left(Y_{i}\right)=\frac{\gamma_{i}^{2}\;var\left(\zeta \right)}{\lambda_{i}^{2}\;var\left(\xi \right)+\gamma_{i}^{2}\;var\left(\zeta \right)+var\left(\varepsilon_{i}\right)}$
Indicator reliability	$Rel\left(Y_{i}\right)=\frac{\lambda_{i}^{2}\;var\left(\xi \right)+\gamma_{i}^{2}\;var\left(\zeta \right)}{\lambda_{i}^{2}\;var\left(\xi \right)+\gamma_{i}^{2}\;var\left(\zeta \right)+var\left(\varepsilon_{i}\right)}$
True score consistency	$Con\left(\tau_{i}\right)=\frac{\lambda_{i}^{2}\;var\left(\xi \right)}{\lambda_{i}^{2}\;var\left(\xi \right)+\gamma_{i}^{2}\;var\left(\zeta \right)}$
True score occasion-specificity	$OSpec\left(\tau_{i}\right)=\frac{\gamma_{i}^{2}\;var\left(\zeta \right)}{\lambda_{i}^{2}\;var\left(\xi \right)+\gamma_{i}^{2}\;var\left(\zeta \right)}$
**MTMS**	
Variance decomposition	$var\left(Y_{i}\right)=var\;\left(\xi_{i}\right)+\gamma_{i}^{2}\;var\left(\zeta \right)+var\left(\varepsilon_{i}\right)$
Indicator consistency	$Con\left(Y_{i}\right)=\frac{var\left(\xi_{i}\right)}{var\left(\xi_{i}\right)+\gamma_{i}^{2}\;var\left(\zeta \right)+var\left(\varepsilon_{i}\right)}$
Indicator occasion-specificity	$\text{OSpec }\left(Y_{i}\right)=\frac{\gamma_{i}^{2}\;var\left(\zeta \right)}{var\left(\xi_{i}\right)+\gamma_{i}^{2}\;var\left(\zeta \right)+var\left(\varepsilon_{i}\right)}\;$
Indicator reliability	$Rel\left(Y_{i}\right)=\frac{var\left(\xi_{i}\right)+\gamma_{i}^{2}\;var\left(\zeta \right)}{var\left(\xi_{i}\right)+\gamma_{i}^{2}\;var\left(\zeta \right)+var\left(\varepsilon_{i}\right)}$
True score consistency	$Con\left(\tau_{i}\right)=\frac{var\left(\xi_{i}\right)}{var\left(\xi_{i}\right)+\gamma_{i}^{2}\;var\left(\zeta \right)}$
True score occasion-specificity	$OSpec\left(\tau_{i}\right)=\frac{\gamma_{i}^{2}\;var\left(\zeta \right)}{var\left(\xi_{i}\right)+\gamma_{i}^{2}\;var\left(\zeta \right)}$
**GSG**	
Variance decomposition	$var\left[Y_{i}\left(t\right)\right]=\lambda_{i}^{2}\;var\left(\xi_{\text{int}}\right)+t^{2}\;\lambda_{i}^{2}\;var\left(\xi_{\text{lin}}\right)+2t\lambda_{i}^{2}\text{ cov}\left(\xi_{\text{int}},\xi_{\text{lin}}\right)+\gamma_{i}^{2}\;var\left(\zeta \right)+var\left(\varepsilon_{i}\right)$
Indicator consistency	$Con\left[Y_{i}\left(t\right)\right]=\frac{\lambda_{i}^{2}\;var\left(\xi_{\text{int}}\right)+t^{2}\;\lambda_{i}^{2}\;var\left(\xi_{\text{lin}}\right)+2t\lambda_{i}^{2}\text{ cov}\left(\xi_{\text{int}},\xi_{\text{lin}}\right)}{\lambda_{i}^{2}\;var\left(\xi_{\text{int}}\right)+t^{2}\;\lambda_{i}^{2}\;var\left(\xi_{\text{lin}}\right)+2t\lambda_{i}^{2}\text{ cov}\left(\xi_{\text{int}},\xi_{\text{lin}}\right)+\gamma_{i}^{2}\;var\left(\zeta \right)+var\left(\varepsilon_{i}\right)}$
Indicator occasion-specificity	$OSpec[Y_{i}\left(t\right)]=\frac{\gamma_{i}^{2}\;var\left(\zeta \right)}{\lambda_{i}^{2}\;var\left(\xi_{\text{int}}\right)+t^{2}\;\lambda_{i}^{2}\;var\left(\xi_{\text{lin}}\right)+2t\lambda_{i}^{2}\text{ cov}\left(\xi_{\text{int}},\xi_{\text{lin}}\right)+\gamma_{i}^{2}\;var\left(\zeta \right)+var\left(\varepsilon_{i}\right)}$
Indicator reliability	$Rel\left[Y_{i}\left(t\right)\right]=\frac{\lambda_{i}^{2}\;var\left(\xi_{\text{int}}\right)+t^{2}\;\lambda_{i}^{2}\;var\left(\xi_{\text{lin}}\right)+2t\lambda_{i}^{2}\text{ cov}\left(\xi_{\text{int}},\xi_{\text{lin}}\right)+\gamma_{i}^{2}\;var\left(\zeta \right)}{\lambda_{i}^{2}\;var\left(\xi_{\text{int}}\right)+t^{2}\;\lambda_{i}^{2}\;var\left(\xi_{\text{lin}}\right)+2t\lambda_{i}^{2}\text{ cov}\left(\xi_{\text{int}},\xi_{\text{lin}}\right)+\gamma_{i}^{2}\;var\left(\zeta \right)+var\left(\varepsilon_{i}\right)}$
True score consistency	$Con\left[\tau_{i}\left(t\right)\right]=\frac{\lambda_{i}^{2}\;var\left(\xi_{\text{int}}\right)+t^{2}\;\lambda_{i}^{2}\;var\left(\xi_{\text{lin}}\right)+2t\lambda_{i}^{2}\text{ cov}\left(\xi_{\text{int}},\xi_{\text{lin}}\right)}{\lambda_{i}^{2}\;var\left(\xi_{\text{int}}\right)+t^{2}\;\lambda_{i}^{2}\;var\left(\xi_{\text{lin}}\right)+2t\lambda_{i}^{2}\text{ cov}\left(\xi_{\text{int}},\xi_{\text{lin}}\right)+\gamma_{i}^{2}\;var\left(\zeta \right)}$
True score occasion-specificity	$OSpec\left[\tau_{i}\left(t\right)\right]=\frac{\gamma_{i}^{2}\;var\left(\zeta \right)}{\lambda_{i}^{2}\;var\left(\xi_{\text{int}}\right)+t^{2}\;\lambda_{i}^{2}\;var\left(\xi_{\text{lin}}\right)+2t\lambda_{i}^{2}\text{ cov}\left(\xi_{\text{int}},\xi_{\text{lin}}\right)+\gamma_{i}^{2}\;var\left(\zeta \right)}$
**ISG**	
Variance decomposition	$var\left[Y_{i}\left(t\right)\right]=var\left(\xi_{{\text{int}}_{i}}\right)+t^{2}\;var\left(\xi_{{\text{lin}}_{i}}\right)+2t\text{ cov}\left(\xi_{{\text{int}}_{i}},\xi_{{\text{lin}}_{i}}\right)+\gamma_{i}^{2}\;var\left(\zeta \right)+var\left(\varepsilon_{i}\right)$
Indicator consistency	$Con\left[Y_{i}\left(t\right)\right]=\frac{var\left(\xi_{{\text{int}}_{i}}\right)+t^{2}\;var\left(\xi_{{\text{int}}_{i}}\right)+2t\text{ cov}\left(\xi_{{\text{int}}_{i}},\xi_{{\text{int}}_{i}}\right)}{var\left(\xi_{{\text{int}}_{i}}\right)+t^{2}\;var\left(\xi_{{\text{int}}_{i}}\right)+2t\text{ cov}\left(\xi_{{\text{int}}_{i}},\xi_{{\text{int}}_{i}}\right)+\gamma_{i}^{2}\;var\left(\zeta \right)+var\left(\varepsilon_{i}\right)}$
Indicator occasion-specificity	$OSpec\left[Y_{i}\left(t\right)\right]=\frac{\gamma_{i}^{2}\;var\left(\zeta \right)}{var\left(\xi_{{\text{int}}_{i}}\right)+t^{2}\;var\left(\xi_{{\text{lin}}_{i}}\right)+2t\text{ cov}\left(\xi_{{\text{int}}_{i}},\xi_{{\text{lin}}_{i}}\right)+\gamma_{i}^{2}\;var\left(\zeta \right)+var\left(\varepsilon_{i}\right)}$
Indicator reliability	$Rel\left[Y_{i}\left(t\right)\right]=\frac{var\left(\xi_{{\text{int}}_{i}}\right)+t^{2}\;var\left(\xi_{{\text{lin}}_{i}}\right)+2t\text{ cov}\left(\xi_{{\text{int}}_{i}},\xi_{{\text{lin}}_{i}}\right)+\gamma_{i}^{2}\;var\left(\zeta \right)}{var\left(\xi_{{\text{int}}_{i}}\right)+t^{2}\;var\left(\xi_{{\text{lin}}_{i}}\right)+2t\text{ cov}\left(\xi_{{\text{int}}_{i}},\xi_{{\text{lin}}_{i}}\right)+\gamma_{i}^{2}\;var\left(\zeta \right)+var\left(\varepsilon_{i}\right)}$
True score consistency	$Con\left[\tau_{i}\left(t\right)\right]=\frac{var\left(\xi_{{\text{int}}_{i}}\right)+t^{2}\;var\left(\xi_{{\text{lin}}_{i}}\right)+2t\text{ cov}\left(\xi_{{\text{int}}_{i}},\xi_{{\text{lin}}_{i}}\right)}{var\left(\xi_{{\text{int}}_{i}}\right)+t^{2}\;var\left(\xi_{{\text{lin}}_{i}}\right)+2t\text{ cov}\left(\xi_{{\text{int}}_{i}},\xi_{{\text{lin}}_{i}}\right)+\gamma_{i}^{2}\;var\left(\zeta \right)}$
True score occasion-specificity	$OSpec\left[\tau_{i}\left(t\right)\right]=\frac{\gamma_{i}^{2}\;var\left(\zeta \right)}{var\left(\xi_{{\text{int}}_{i}}\right)t^{2}\;var\left(\xi_{{\text{lin}}_{i}}\right)+2t\text{ cov}\left(\xi_{{\text{int}}_{i}},\xi_{{\text{lin}}_{i}}\right)+\gamma_{i}^{2}\;var\left(\zeta \right)}$

Rel, reliability coefficient; Con, consistency coefficient; OSpec, occasion-specificity coefficient.

## LGC models

In contrast to LST models, which represent reversible short-term fluctuations in behavior, LGC models account for long-lasting trait-change processes such as, for example, enduring changes in height, intelligence, or anxiety. Growth models typically include a continuous latent factor that represents true individual differences at a particular time point (often participants' true initial trait scores) and is often called *intercept factor*. In addition, LGC models usually feature one or more continuous latent factors that represent individual differences in trait change over time, the so-called latent slope, shape, or curve factors. Depending on the hypotheses of a researcher, the slope factor(s) can represent, for example, linear, quadratic, cubic, or an unspecified form of latent trait change.

In this paper, we focus on linear growth. Extensions to non-linear LGC models are straightforward in principle, as discussed later on. In LGC models, the focus is typically on the separation of measurement error from true individual differences in initial trait and trait change as well as on the estimation of the “growth parameters,” such as means, variances, and covariances of intercept (initial trait) and slope (trait change) factors. In addition, covariates or outcomes of change may also be included in the model.

### Single-indicator LGC models

**Figure 2:**
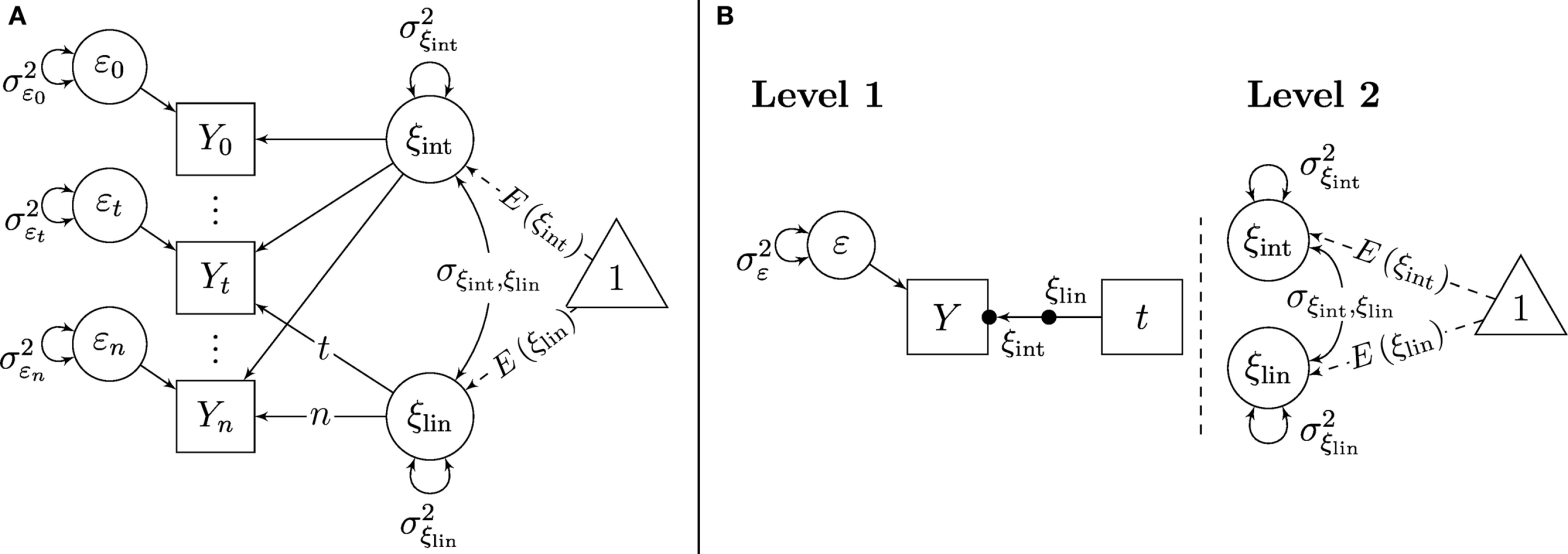
**Single-indicator linear growth model. (A)** Specification as SL-SEM model. **(B)** Specification as ML-SEM model.

Even though the focus of the present paper is on multiple-indicator LGC models, we begin our presentation with a single-indicator LGC model, given that single-indicator LGC models are likely to be somewhat more familiar to many readers. Figure 2 shows path diagrams of the SL-SEM and ML (random coefficient) regression parameterizations of a single-indicator linear LGC model. Figure 2A shows that in the SL-SEM specification of this model, there is a single observed variable *Y_t_* at each time point. The variables *Y_t_* are regressed on an intercept factor ξ_int_ ≡ ξ_0_, which represents the latent trait scores at time 0 and a linear slope factor ξ_lin_ ≡ (ξ_1_ − ξ_0_), which represents the latent difference between the trait scores at time 1 and the trait scores at time 0 (for details on the definition of these factors on the basis of concepts of LST theory, see Geiser et al., 2013):

(7) $$Y_{t}=\xi_{\text{int}}+(t)\xi_{\text{lin}}+\varepsilon_{t},$$

where *t* indicates the linear effect of time on the trait values (*t* = 0, …, *s*, …, *n*). That is, for the first time point (*t* = 0), the slope (or trait change) factor does not have an effect on the indicator *Y_t_*, for the second time point (*t* = 1), the effect is 1, for the third time point (*t* = 2), the effect is 2 and so on. Note that non-linear change could be modeled by including a different “version” of time, for example *t*^2^ for quadratic trait change. Intercept and slope factor can be correlated as shown by the covariance parameter σ _ξ_int_, ξ_lin__. Measurement error and situation-specific variability are reflected in the error variables ε_*t*_ (One of the limitations of single-indicator LGC models is that measurement error cannot be separated from true state variability).

Figure 2B shows the ML parameterization of the single-indicator LGC model. Note that in contrast to the LST models shown in Figure 1, LGC models include both, random intercept and random slope parameters at Level 1. This is because in LGC models, it is assumed that (1) there is a non-zero effect of time on the outcomes and (2) that the effect of time can vary across individuals (although the functional form of change is assumed to be the same for all individuals, e.g., linear change).

Because of the presence of random slopes, an additional symbol is needed in the ML-SEM path diagrams for LGC models in order to depict the random slopes in the Level-1 part of the models. We again follow the conventions proposed by Muthén and Muthén (1998–2012) and depict random slopes in terms of a dot (•) in the middle of the arrow pointing from the time variable to the indicators and labeling the slopes with the relevant latent variable name (here ξ_lin_). In the same way as the random intercepts ξ_int_, the random slopes ξ_lin_ can be conceived of as latent variables whose parameters (means, variances, and covariances) are modeled on Level 2 (the person level). This is because not only the intercepts, but also the slopes of the growth curves can vary across individuals (Level-2 units) in LGC models. In LGC models, the random intercepts represent the latent trait levels at the onset of the study (i.e., at time 0), whereas the random slopes represent linear trait change.

In terms of the ML modeling framework, single-indicator LGC models can be seen as random coefficient regression models (e.g., Luke, 2004) with a single outcome variable (*Y_t_*) that is regressed on the single Level-1 predictor *time*. The intercepts and slopes of this regression are random coefficients whose means and variances are Level-2 parameters. Non-time-varying covariates of the intercept and slope would be modeled as Level-2 predictors in the ML parameterization of this model. Given the fact that single-indicator LGC models use only a single outcome variable if specified within the ML framework, computer software for conventional ML regression analyses such as SPSS or HLM could be used to estimate the parameters of the model. This is different for LST and multiple-indicator LGC models, which use *multiple* outcomes at Level 1 and thus require a ML-SEM approach and more specialized software such as, for example, Mplus (Muthén and Muthén, 1998–2012).

### Multiple-indicator LGC models

**Figure 3:**
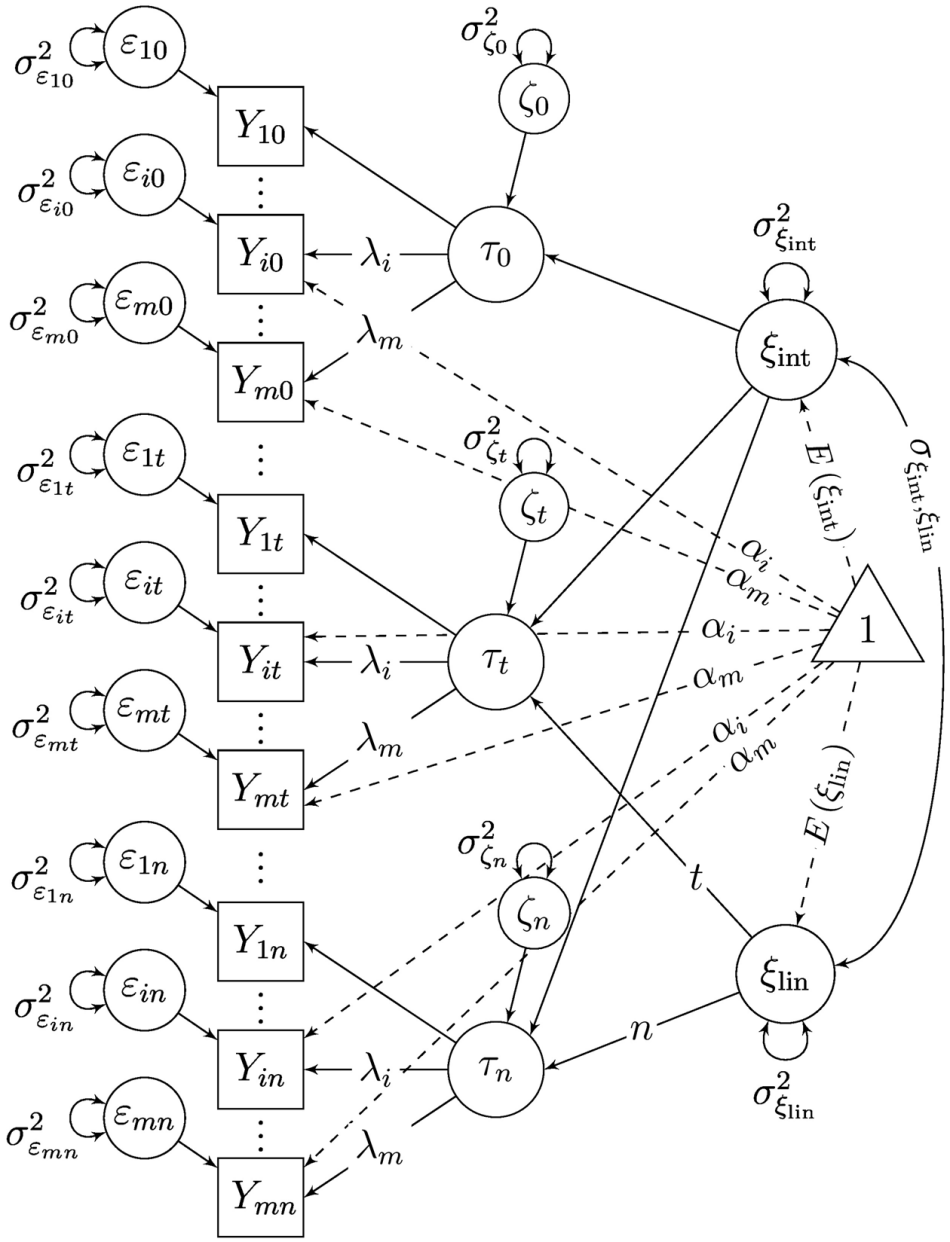
**Linear second-order growth model**. SL-SEM model.

The second-order LGC model (SGM; McArdle, 1988) shown in Figure 3 is probably the most widely known multiple-indicator LGC model to date. In this model, a time-specific latent state variable τ_*t*_ is included as a first-order factor at each time point to separate measurement error from true individual differences at each time point (Sayer and Cumsille, 2001). The trait-change process is modeled in terms of second-order intercept and slope factors. The time-specific residual factors ζ_*t*_ have the same meaning as the latent state residual factors in STMS and MTMS models and capture state-variability processes around the growth curves (Geiser et al., 2013).

**Figure 4:**
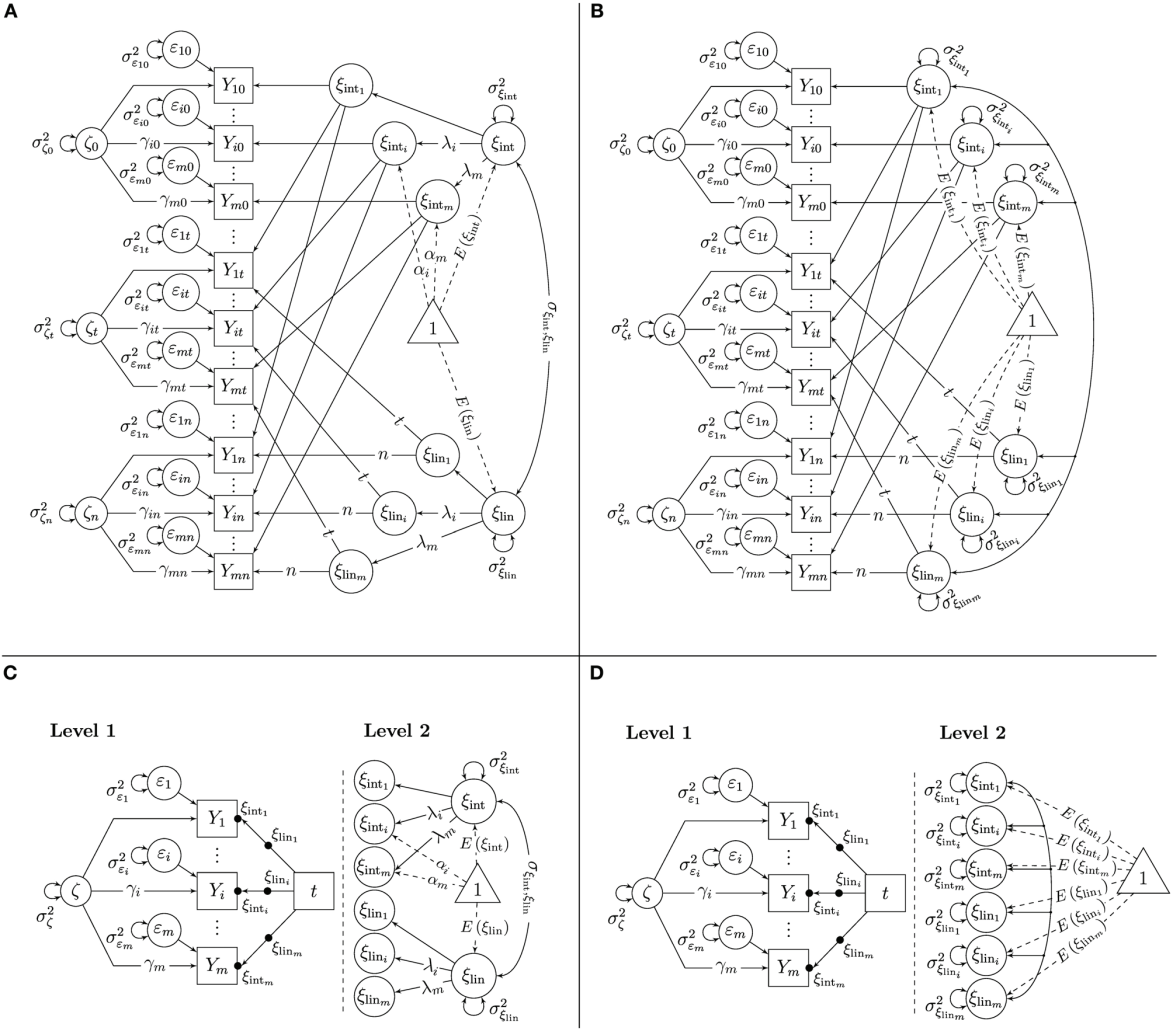
**Multiple-indicator linear growth models. (A)** GSG model as SL-SEM model. **(B)** ISG model as SL-SEM model. **(C)** GSG model as ML-SEM model. **(D)** ISG model as ML-SEM model.

In this article, we discuss two alternative multiple-indicator LGC models that relax some restrictive assumptions of the SGM and that are more easily specified within the ML-SEM framework than the SGM. The SL-SEM versions of these models as well as their relationship to McArdle's SGM are described in more detail in Eid et al. (2012) as well as Bishop et al. (submitted). Like the SGM, the *generalized second-order growth model* (GSGM; Figures 4A,C) assumes that all indicators share the same trait-change process within scaling differences; however, the GSGM relaxes an implicit restriction made in the SGM, according to which the factor loadings λ_*i*_ on the latent state residual factors ζ_*t*_ are equal to the loadings on the intercept factor ξ_int_ (Bishop et al., submitted). The *indicator-specific growth model* (ISGM; Figures 4B,D) additionally relaxes the assumption that all indicators share the same trait-change process within scaling differences made in both the SGM and GSGM and allows each indicator to have its own unique growth trajectory.

### The generalized second-order growth model (GSGM)

In the linear GSGM, it is assumed that the observed variables *Y*_*it*_ are a function of a constant variable-specific intercept parameter α_*i*_ that is time-invariant for the same indicator, a common latent trait factor at time 0 that serves as latent intercept factor ξ_int_ ≡ ξ_0_, a common latent trait difference factor that serves as linear slope factor ξ_lin_ ≡ (ξ_1_ − ξ_0_), a common latent state residual factor ζ_*t*_ that is shared by all observed variables that are measured at the same time point, and a variable-specific measurement error variable ε_*it*_:

(8) $$Y_{it}=\alpha_{i}+\lambda_{i}\xi_{\text{int}}+\lambda_{i}(t)\xi_{\text{lin}}+\gamma_{i}\zeta_{t}+\varepsilon_{it},$$

where *t* again indicates the linear effect of time. The parameters λ_*i*_ and γ_*i*_ represent constant time-invariant scaling parameters (factor loadings). As in the STMS model, these parameters as well as the intercepts α_*i*_ are included in the model to allow for potential differences in scaling between different indicators. Note that in contrast to the SGM, the loadings on the state residual factors ζ_*t*_ are independent model parameters (γ_*i*_) that do not have to be equal to the trait-related loadings λ_*i*_. Time-invariance of factor loadings and intercepts is required in the GSGM to ensure that trait changes over time [as captured by ξ_lin_] can be meaningfully interpreted and are not confounded with changes in the measurement scale.

The common latent intercept factor ξ_int_ reflects individuals' trait levels on the first measurement occasion. The common latent slope factor reflects individuals' rate of linear change in trait scores across time. The common latent state residual factors capture the effects of the situations and/or of person-by-situation interactions as in the STMS, MTMS, and SGM models.

The GSGM can be identified by setting all factor loading parameters of one reference indicator (e.g., *Y*_1*t*_) to 1 (i.e., λ_1_ = γ_1_ = 1) and the intercepts of the same indicator to 0 (i.e., α_1_ = 0). Then, the remaining intercepts, loadings, the variances of all latent variables, and the mean of the latent growth factors, *E*(ξ_int_) and *E*(ξ _lin_) as well as their covariance, are identified and can be estimated as free parameters as long as *m* ≥ 3 and *n* ≥ 3 (For *m* = 2, the model can be identified by additionally setting all γ_*i*_ = 1 for all *i* or by allowing each latent state residual factor to be correlated with at least one external variable in the model). Figure 4A shows a path diagram of the SL-SEM version of the GSGM with the above-mentioned identifying constraints on the loadings and intercepts.

### The indicator-specific growth model (ISGM)

Similar to the MTMS model in LST modeling, the ISGM allows us to relax the assumption of perfectly unidimensional indicators in the context of latent growth modeling. In the linear ISGM, it is assumed that each observed variable *Y_*it*_* is a function of an indicator-specific latent intercept factor ξ_int_*i*__ ≡ ξ_*i*0_, an indicator-specific latent slope factor ξ_lin_*i*__ ≡ (ξ _*i*1_ − ξ _*i*0_), a common latent state residual variable ζ_*t*_ that is shared by all observed variables that are measured at the same time point, and a variable-specific measurement error variable ε_*it*_:

(9) $$Y_{it}=\xi_{{\text{int}}_{i}}+(t)\xi_{{\text{lin}}_{i}}+\gamma_{i}\zeta_{t}+\varepsilon_{it}.$$

Note that the intercept and slope factors in the ISGM are indicator-specific. This implies that indicators are not only allowed to differ in scaling, but also with respect to the initial trait level and rate of trait change. Linear change is again assumed by setting the loadings on the slope factors to *t* = 0, …, *s*, …, *n*. The common latent state residual factors capture the effects of the situations and/or of person-by-situation interactions as in the previously discussed models.

The ISGM can be identified by setting the latent state residual factor loading parameters of one reference indicator (e.g., *Y*_1*t*_) to 1 (i.e., γ_1_ = 1). Furthermore, all factor loadings on the intercept factors have to be set to 1 in the ISGM (there is an implicit coefficient of one before ξ_int_*i*__ in Equation 9), and all factor loadings on the slope factors have to be set to *t*. There are no additive constants in the equation, which means that all constant intercepts parameters have to be fixed to zero. Then, the remaining state residual factor loadings, the variances of all latent variables, the means of the latent intercept and slope factors as well as their covariances are identified and can be estimated as free parameters as long as *m*≥ 3 and *n*≥ 3 (For *m* = 2, the model can be identified by additionally setting all γ_*i*_ = 1 for all *i* or by allowing each latent state residual factor to be correlated with at least one external variable in the model).

Non-linear growth trajectories could again be tested by using different versions of the time variable. Note that the form of change need not be the same for different indicators in the ISGM. For example, change could be linear for one indicator and quadratic for another one. Figure 4B shows a path diagram of the SL-SEM specification of the ISGM with the above-mentioned identifying constraints on the loadings and intercepts.

### The GSGM and ISGM as ML-SEM models

In the ML-SEM parameterization of the GSGM and ISGM, the effect of time is modeled in terms of a multivariate random coefficient regression analysis that includes both random intercepts and random slopes. *Multivariate* here means that in contrast to single-indicator LGC models, we are now dealing with multiple outcome variables simultaneously. The indicator-specific random intercepts ξ_int_*i*__ and random slopes ξ_lin_*i*__ are again treated as latent variables whose parameters (means, variances, and covariances) are modeled on Level 2 (the person level).

The ML-SEM representation of the GSGM is illustrated in Figure 4C. The left panel of Figure 4C shows the Level-1 part of the GSGM. As in LST models, on Level 1, three different effects are modeled: (1) the effect of measurement error as represented by the time-invariant error variance parameters Var(ε_*i*_), the effect of the common latent state residual factor that is shared between indicators within scaling differences as represented by the time-invariant variance parameter *Var*(ζ) and the time-invariant factor loadings γ_*i*_, and (3) the effect of time, as represented by the random intercepts ξ_int_*i*__ and the random slopes ξ_lin_*i*__. Note that in contrast to LST models, the effect of time in LGC models includes not only random intercepts, but also random slopes.

On Level 2, the means, variances, and covariances of the random intercepts and slopes are modeled. In the GSGM, it is assumed that all random intercepts are linear functions of a common intercept factor within scaling differences, and that all random slopes are linear functions of a common slope factor within scaling differences (see the right panel of Figure 4C). Therefore, on Level 2, we estimate constant intercepts α_*i*_ and factor loadings λ_*i*_ as scaling parameters, as well as the means, variances, and the covariance of the common growth factors. Note that the scaling parameters λ_*i*_ on the intercept and slope factors are identical for the same indicator, reflecting part of the “measurement equivalence across time” assumption. Therefore, an equality constraint on these loadings has to be implemented when estimating the parameters of the model^3^.

In contrast to the GSGM, the ISGM uses indicator-specific growth factors (see Figure 4D for the ML-SEM representation of this model). Therefore, no common factors are introduced on Level 2 in this model. On Level 2, we simply model the means, variances, and covariances of the indicator-specific growth factors. The Level-1 part of the ISGM is the same as in the STMS, MTMS, and GSGM models.

### Variance components and coefficients in the GSGM and ISGM

Variance components as well as the coefficients of *Con*, *OSpec*, and *Rel* can also defined in the GSGM and ISGM (for additional coefficients in these models see Eid et al., 2012). Table 1 shows the corresponding equations. It can be seen that the *Con* coefficient in LGC models includes both intercept-trait variance and growth (trait-change) variance. The definition of the *OSpec* and *Rel* coefficients is similar as in the STMS and MTMS models.

## Application of LST and multiple-indicator growth models as ML-SEM models

As mentioned previously, the analysis of LST and multiple-indicator LGC models within the ML-SEM framework is especially useful and flexible when there are a large number of individually varying and/or unequally spaced time points and/or when there is missing data. EMA studies can be seen as a prototypical example, because they are typically prone to the above issues: People are often repeatedly prompted to answer questions on a large number of randomly chosen, individually-varying times. The time-intervals are typically not equally spaced, and they often also are not the same for all individuals in the study. In addition, missing values are common in these studies due to, for example, people (randomly or non-randomly) not responding to prompts, technical problems, or permanent drop-out of the study. Below we present an illustrative application of LST and LGC models to EMA data of adolescents' positive mood using the ML-SEM specification of these models.

### Methods

#### Sample and measures

The data analyzed for this example come from a study using EMA of adolescents' social, environmental, and emotional cues of eating (Grenard et al., 2013). Participants included 158 adolescents (mean age = 15.97; 57% female) who were recruited from economically disadvantaged high schools in Southern California. For seven days, each youth carried a handheld electronic device on which they responded to questions related to characteristics of the environment (i.e., what was happening, where, and with whom), their internal mood states, and what they were eating.

Responses were both randomly prompted and user-initiated; user-initiated responses were given when the participant ate a snack or meal (for the sake of simplicity, and given that our focus was on the presentation of statistical modeling rather than substantive issues, we did not distinguish between random prompts and user-initiated responses in the present example; however, in an actual substantive study, this distinction may be important). A total of 3,992 momentary assessments were recorded.

For the purposes of the present illustration of how to analyze LST and LGC models as ML-SEM models, we selected three repeatedly administered items referring to adolescents' positive mood (“Were you feeling happy/energetic/cheerful?”). Each item was rated on a scale ranging from 0 to 100, with larger scores indicating higher levels of each dimension of positive mood. In the present analysis, we treated each item as a continuous variable, given the large number of intervals on the response scale. The items showed model-based reliability estimates between.61 and.75 in the present analyses (happy:.69–.71; energetic:.61–.63; cheerful:.75; estimates varied slightly across models; see the Results section for more details). Given that the estimates were for single-item measures, the reliabilities can be seen as good.

The present application is an interesting case for the application of LST and LGC models. On the one hand, mood states are classical state-variability constructs, so that one may expect LST models (STMS or MTMS) to fit well and ask the question of why trait-change models (GSGM or ISGM) would be needed for these data. On the other hand, it has been shown that the day of week can have an influence on mood levels, with a significant and close-to-linear increase in mood from Monday to Saturday (Csikszentmihalyi and Hunter, 2003). Therefore, an LST model may not be sufficient for the present data, as there may have been more than just momentary (short-term) changes in mood levels over the course of the study as well, making the use of a growth model necessary.

Whereas in the STMS and GSGM models, the three items were treated as indicators of a single trait-mood construct, the MTMS and ISGM models allowed for item-specific traits. Hence, with our analyses, we were able to also test whether the three items measured a unidimensional mood construct or distinct (albeit potentially related) facets of positive mood.

**Figure 5:**
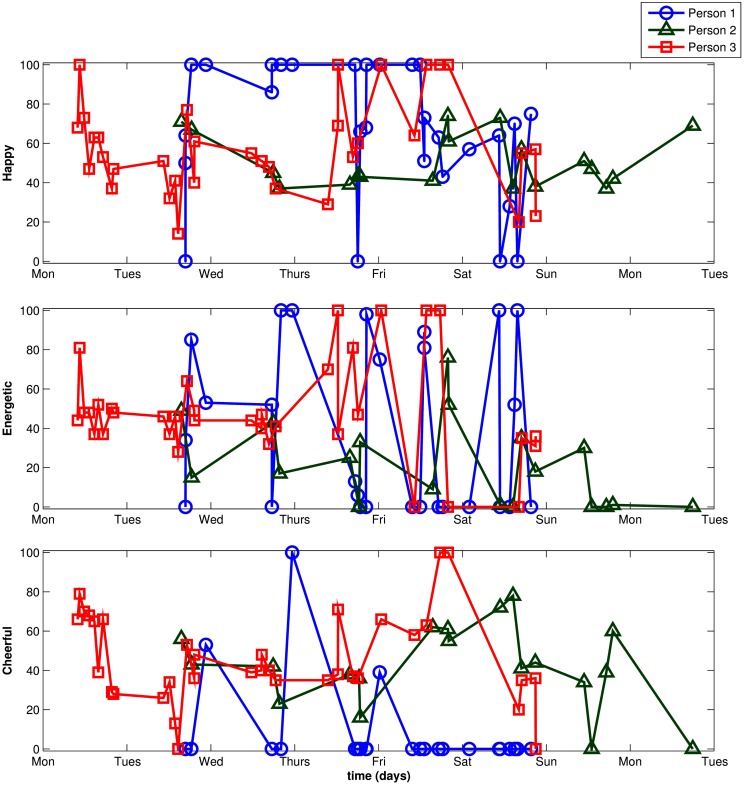
**Raw scores for three randomly selected individuals**.

The analyses reported below are based on a total of 3,712 available observations for the three mood items (average number of time points per individual: 23.49, minimum = 7, maximum = 56). Figure 5 shows the observed item scores of three randomly selected individuals from the sample. Figure 5 illustrates typical issues in longitudinal EMA data that make the use of an ML approach appealing: many observations for each individual, unequally-spaced times of observation within and between individuals, and the presence of missing data.

We fit the ML-SEM versions of the STMS (Figure 1C), MTMS (Figure 1D), GSGM (Figure 4C), and ISGM (Figure 4D) models to the three items of the mood questionnaire using the TYPE = TWOLEVEL option in the computer program Mplus 7 (Mplus scripts for all four models are provided in the Appendix). A person ID variable identifying each individual served as the cluster variable to separate Level 1 (the 3,712 measurements) from Level 2 (the 158 individuals) in the ML-SEMs. A time variable was included as Level-1 predictor as shown in Figures 1, 4. The time variable reflected time measured in days. For most participants, the first assessment started on a Monday. Thus, we set the time variable's zero point (*t* = 0) to Monday. This means that parameters related to the intercept factor in the GSGM and ISGM (i.e., the intercept factor means, variances, and covariances) refer to participants' latent trait levels on the first Monday of the study.

### Results

Table 2 shows the maximum-likelihood estimated descriptive statistics for the three items for Level 1 and Level 2. The intraclass correlations for the three items ranged between.32 and.36, indicating substantial consistency (trait effects) of the item responses across time. Table 3 provides model fit information for the four models estimated for these data.

**Table 2:** **Estimated means, standard deviations, covariances, and correlations for the mood items used in the application**.

	**Indicator**	***Y*_1_**	***Y*_2_**	***Y*_3_**
**LEVEL 1**				
	*SD*	21.611	23.424	22.553
	*Y*_1_	–	239.512	276.931
	*Y*_2_	0.473	–	267.473
	*Y*_3_	0.568	0.506	–
**LEVEL 2**				
	M	61.935	47.153	51.491
	*SD*	15.269	16.203	16.814
	*Y*_1_	–	185.758	221.915
	*Y*_2_	0.751	–	217.321
	*Y*_3_	0.864	0.798	–

*Correlations are shown below the diagonal, covariances are shown above the diagonal. Y_1_, Happy; Y_2_, Energetic; Y_3_, Cheerful. Values are based on Mplus maximum likelihood estimates*.

**Table 3:** **Goodness of fit statistics for different models**.

**Model**	**χ^2^(*df*)**	***p*(χ^2^)**	**RMSEA**	**SRMR**		**CFI**	**AIC**	**BIC**
				**Level 1**	**Level 2**			
STMS	525.23 (6)	<0.001	0.16	0.05	0.14	0.75	79726.20	79799.68
MTMS	26.15 (3)	<0.001	0.05	0.04	0.00	0.99	79233.12	79324.97
GSG							79613.56	79705.41
ISG							**79106.94**	**79309.02**

*N = 158 Participants, 3,712 Observations. RMSEA, Root mean square error of approximation; SRMR, Standardized root mean square residual; CFI, Comparative fit index; AIC, Akaike information criterion; BIC, Bayesian information criterion; STMS, Single-trait multi-state; MTMS, Multi-trait multi-state; GSG, Generalized second-order growth; ISG, Indicator-specific growth. Lowest AIC and BIC values are printed in bold face*.

Whereas the STMS and MTMS models did not require the inclusion of random slopes in Mplus, the GSGM and ISGM did require the modeling of random slopes, because the latter models included growth factors that measure trait change across time. The Mplus option TYPE = TWOLEVEL RANDOM allows for the specification of random slope coefficients on Level 1. Note that a global chi-square test of model fit was only available for the STMS and MTMS models, but not for the GSGM and ISGM, because Mplus 7 does not provide chi-square statistics under the option TWOLEVEL RANDOM.

The model fit statistics indicated that the STMS model, which includes only a single general trait (i.e., a single random intercept) factor for all indicators at Level 2, did not fit the data well (see Table 3). The less restrictive MTMS model relaxes the assumption that all indicators measure the exact same trait, but still assumes that there are only short-term fluctuations and no trait changes across time. The MTMS model fit the data better. According to common standards for approximate model fit (e.g., Hu and Bentler, 1999; Schermelleh-Engel et al., 2003), the fit could be seen as acceptable. Nonetheless, the χ^2^ test indicated significant misfit (*p* < 0.001). The three *df* in the MTMS model are a result of the three fixed-to-zero coefficients of time in this model. Hence, the fact that the MTMS model showed some misfit might indicate that there is a significant effect of time in these data, meaning that a pure state-variability process may not be sufficient to explain the observed data structure. In other words, there may have been trait change in mood in addition to state fluctuations.

The GSGM and ISGM allow for a (linear) effect of time that can vary across individuals at Level 2 (random slope) in addition to state-variability at Level 1. Although these models could not be evaluated via the χ^2^ test of model fit or approximate fit indices, information criteria (IC) were still available for these models. The Akaike's information criterion (AIC) and Bayesian information criterion (BIC) values for all four models are shown in Table 3. The AIC and BIC values allowed us to compare the fit of the GSGM and ISGM to the fit of the STMS and MTMS models. It can be seen that both coefficients favored (were lowest for) the ISGM, indicating that a model with a linear trait-change component fit these data somewhat better than a model that assumes a pure state-variability process. Below we report the parameter estimates of the two best fitting models (MTMS and ISGM) for illustrative purposes.

Table 4 shows the unstandardized maximum likelihood parameter estimates and standard errors for the MTMS model (left panel) and the linear ISGM (right panel). In both the MTMS and ISGM, situational and/or person × situation effects were substantial and of similar magnitude, as shown by the large and significant variance of the latent state residual factor in each model (and the substantial *OSpec* coefficients for each item, see discussion below). The estimated intercept factor means in the ISGM indicated a mean latent trait level of positive mood between 42.14 (for the item *energetic*) and 58.65 (for the item *happy*) on the first Monday of the study. Except for happy, these values indicated mood levels below the middle point (50) of the response scale, potentially reflecting a “Monday low,” especially for *energetic*. The estimated trait means in the MTMS model were higher, which makes sense, because the values in this model indicate a type of average *across the entire week* (the MTMS model assumes a stable trait level across time).

**Table 4:** **Parameter estimates and standard errors for the MTMS and ISG Models**.

**Parameter Label**	**Parameter**	**MTMS Model**		**ISG Model**	
		**Estimate**	***SE***	**Estimate**	***SE***
**LEVEL 1**					
State residual	γ_1_	1.00	–	1.00	–
factor loadings	γ_2_	0.97	0.04	1.01	0.04
	γ_3_	1.12	0.04	1.11	0.05
State residual factor *v*ariances	*v*ar(ζ)	248.30	13.87	219.42	13.22
Error *v*ariances	*v*ar (ε_1_)	218.81	10.44	209.01	10.18
	*v*ar (ε_2_)	317.60	11.99	305.64	12.28
	*v*ar (ε_3_)	199.33	11.99	205.71	11.65
**LEVEL 2**					
Factor means	*E*(ξ_1_) or *E*(ξ_int__1_)	61.92	1.28	58.65	2.11
	*E*(ξ_2_) or *E*(ξ_int__2_)	47.13	1.38	42.14	1.94
	*E*(ξ_3_) or *E*(ξ_int__3_)	51.47	1.42	46.72	2.17
	*E*(ξ_lin__1_)	–	–	0.72	0.34
	*E*(ξ_lin__2_)	–	–	1.10	0.32
	*E*(ξ_lin__3_)	–	–	1.03	0.36
Factor *v*ariances	*v*ar(ξ_1_) or *v*ar(ξ_int__1_)	232.65	29.19	529.85	82.15
	*v*ar(ξ_2_) or *v*ar(ξ_int__2_)	261.80	34.51	349.67	71.71
	*v*ar(ξ_3_) or *v*ar(ξ_int__3_)	282.47	36.09	518.73	87.47
	*v*ar(ξ_lin__1_)	–	–	11.92	2.32
	*v*ar(ξ_lin__2_)	–	–	5.75	1.94
	*v*ar(ξ_lin__3_)	–	–	10.80	2.40

*Entries represent unstandardized parameter estimates. Dashes indicate fixed parameters for which no standard errors are computed or parameter type not applicable, i = 1 Happy; i = 2, Energetic; i = 3 Cheerful*.

**Figure 6:**
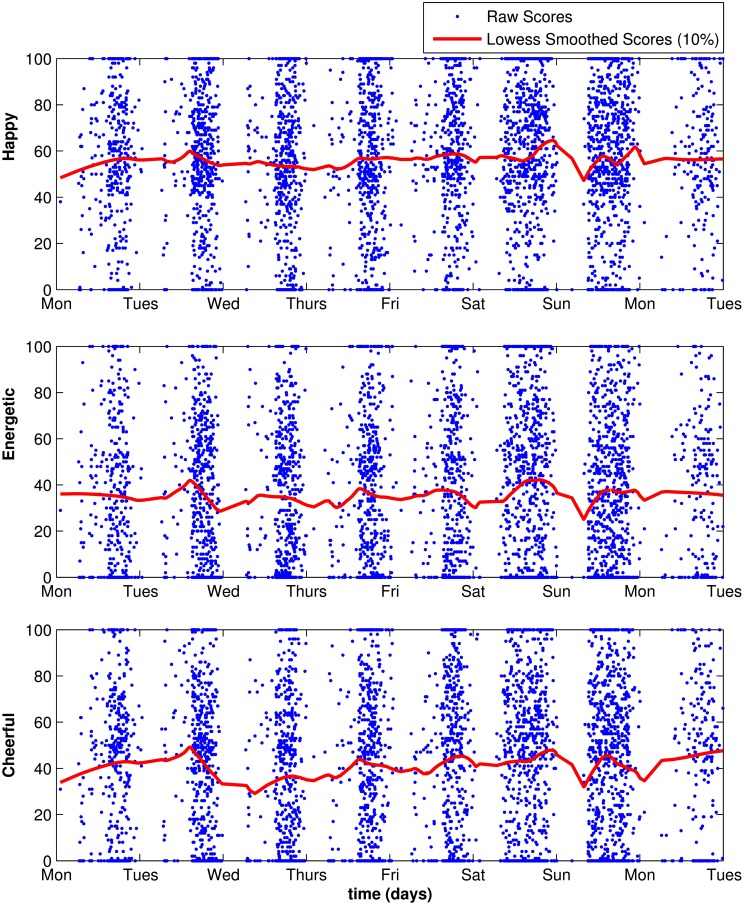
**Lowess fit (local regression using weighted least squares and a first degree polynomial) using scores for all individuals**.

All three slope factor variances estimated in the ISGM were statistically significant (*p* < 0.001), indicating that there were significant individual differences in linear trait change for all three items. Furthermore, the means of the three slope factors were positive and significantly different from zero for all three items. This indicated a slight increase in mood over the course of the study, that is, from Monday to Saturday. For example, the estimated average linear increase in the trait level for *energetic* was 1.10 units on the response scale per day across the week. Figure 6 illustrates the trends in the raw data in terms of lowess-fitted curves.

Table 5 shows the estimated covariances and correlations in the MTMS model. It can be seen that although the indicator-specific trait factors in the MTMS model were strongly correlated, all correlations were substantially smaller than 1. This explains why the STMS model with a single trait factor did not fit these data well. We may conclude that although there was substantial convergent validity between the items at the latent trait level, each item still measured a slightly different facet of positive trait mood.

**Table 5:** **Estimated covariances and correlations between indicator-specific trait factors in the MTMS model**.

	**ξ_1_**	**ξ_2_**	**ξ_3_**
ξ_1_	—	185.08 (27.50)	221.45 (29.76)
ξ_2_	0.75 (0.04)	—	216.56 (31.18)
ξ_3_	0.86 (0.03)	0.80 (0.04)	—

*Correlations are shown below the diagonal, covariances are shown above the diagonal. Standard errors are given in parentheses*.

Similar conclusions can be drawn from the growth factor correlations in the ISGM (see Table 6). Again, the intercept and slope factors were highly, but not perfectly correlated, indicating that the item true scores showed similar, albeit partly distinct growth processes. In both the MTMS and ISGM models, the lowest correlations were found between the trait/trait change components of *happy* and *energetic*, implying that these items showed the strongest level of discriminant validity (or the lowest level of convergent validity, depending on the researcher's point of view). The highest correlations were found between the *happy* and *cheerful* traits, indicating that these items were measuring strongly related facets of mood. Correlations between intercept and slope factors in the ISGM were consistently negative, indicating that individuals with lower initial trait scores had larger change scores than individuals with higher initial trait scores.

**Table 6:** **Estimated covariances and correlations between indicator-specific growth factors in the MTMS model**.

	**ξ_int_1__**	**ξ_int_2__**	**ξ_int_3__**	**ξ_lin_1__**	**ξ_lin_2__**	**ξ_int_3__**
ξ_int_1__	–	317.10 (64.75)	462.28 (75.60)	−59.11 (12.45)	−25.23 (9.96)	−49.32 (11.51)
ξ_int_2__	0.74 (0.07)	–	362.32 (68.03)	−28.48 (9.91)	−22.47 (10.38)	−28.80 (10.05)
ξ_int_3__	0.88 (0.04)	0.85 (0.06)	–	−50.29 (11.51)	−34.00 (10.37)	−49.87 (12.83)
ξ_lin_1__	−0.74 (0.05)	−0.44 (0.11)	−0.64 (0.08)	–	5.62 (1.73)	10.57 (2.08)
ξ_lin_2__	−0.46 (0.13)	−0.50 (0.13)	−0.62 (0.12)	0.68 (0.11)	–	6.93 (1.79)
ξ_lin_3__	−0.65 (0.08)	−0.47 (0.12)	−0.67 (0.07)	0.93 (0.05)	0.88 (0.09)	–

*Correlations are shown below the diagonal, covariances are shown above the diagonal. Standard errors are given in parentheses*.

Table 7 shows the *Con*, *OSpec*, and *Rel* coefficients that were computed based on the parameter estimates for both models. According to the MTMS model, between 61 and 75% of the observed variability in the item scores represented true score variance (*Rel* coefficients). Moreover, about 50% of each item's true score variance reflected stable individual differences (trait effects) in this model, whereas the remaining 50% were due to the situational and/or person × situation interaction effects.

**Table 7:** **Estimated coefficients in the MTMS and ISG models**.

**MTMS model**						**ISG model^a^**				
***i***	***Con*(*Y_i_*)**	***OSpec* (*Y_i_*)**	***Rel* (*Y_i_***	***Con* (τ_*i*_)**	***OSpec* (τ_*i*_)**	***Con* (*Y_i_*)**	***OSpec* (*Y_i_*)**	***Rel* (*Y_i_*)**	***Con* (τ_*i*_)**	***OSpec* (τ_*i*_)**
1	.33	.35	.69	.48	.52	.42	.30	.71	.58	.42
2	.32	.28	.61	.53	.47	.36	.27	.63	.57	.43
3	.36	.39	.75	.48	.52	.42	.33	.75	.56	.44

*Rel, reliability coefficient; Con, consistency coefficient; OSpec, occasion-specificity coefficient; i = 1, Happy; i = 2, Energetic; i = 3, Cheerful*.

a *Values shown for ISG model represent the average over the entire time interval for which data was present*.

**Figure 7:**
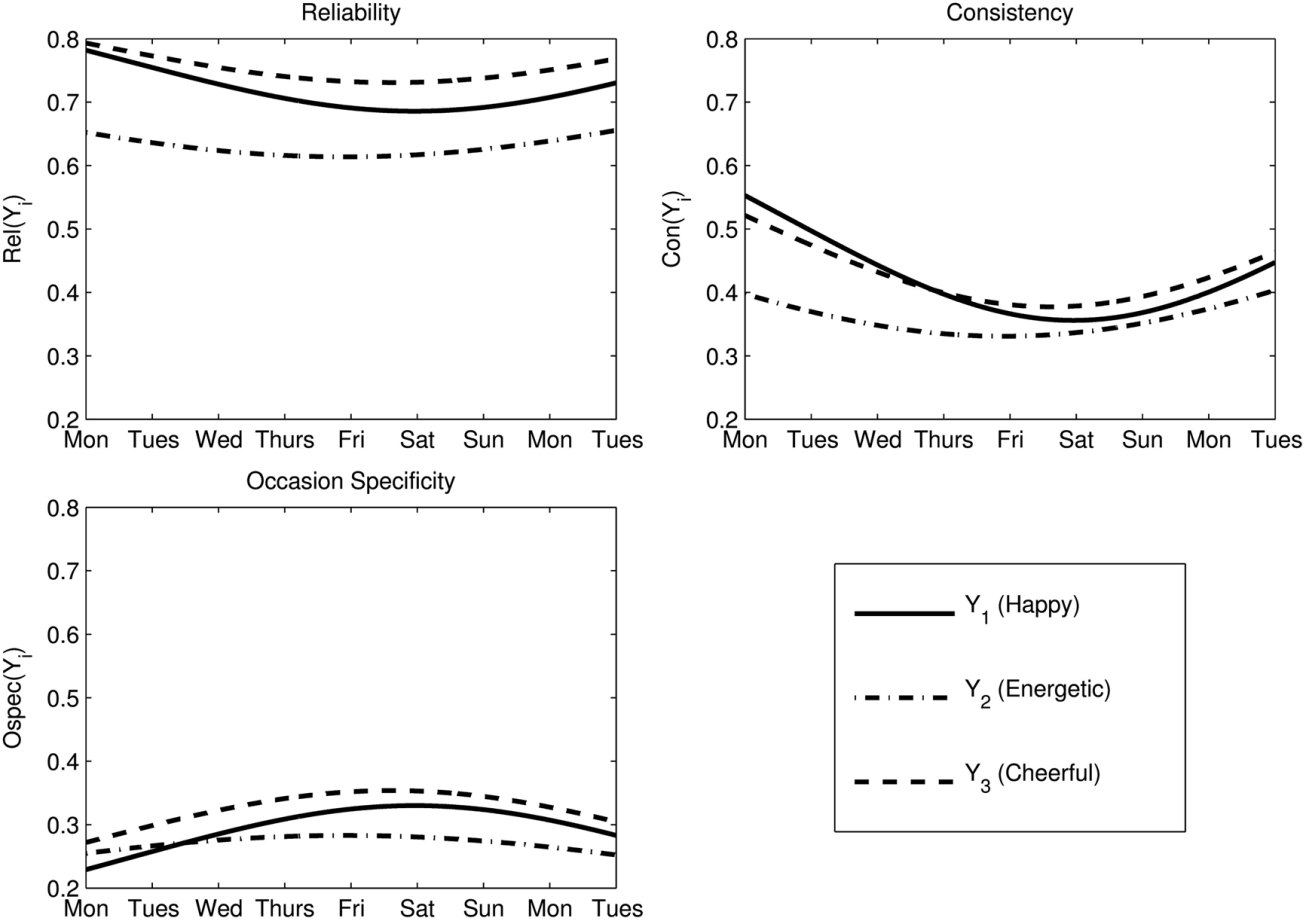
**Model-based coefficients for the ISG model**. *i* = 1: Happy, *i* = 2: Energetic, *i* = 3: Cheerful.

Note that the coefficient estimates in the ISGM vary across time given the non-zero effect of time in this model (see formulas in Table 1). Therefore, we present the *average* coefficient estimates for the ISGM in Table 7. The full range of coefficient estimates for the duration of the study is provided in Figure 7. Table 7 shows that the average coefficients for the ISGM were similar to the coefficient estimates based on the MTMS model, except that the *Con* estimates were slightly higher, and the *OSpec* estimates were slightly lower in the ISGM compared to the MTMS model. This is most likely due to the fact that the ISGM, but not the MTMS model, accounted for trait change over time. The trait-change component is viewed as part of the trait consistency in the ISGM (see formulas in Table 1), leading to an increase in consistency estimates relative to occasion-specificity in the case of a significant trait-change variance in this model.

#### Summary of findings

In summary, our ML-SEM results for the present data set indicated that the three mood items measured strongly related, albeit statistically distinct aspects of trait mood and/or changes in trait mood over time. This makes sense, given that, for example, the item *energetic* involves an arousal component, which is not shared with the *happy* or *cheerful* items. Furthermore, the analyses revealed that each of the three items measured both trait and state components of individuals' mood. Roughly 50% of the true variability was attributable to stable traits (i.e., consistency) whereas the remaining 50% were due to situation-specific influences or person × situation interactions (i.e., state-variability). According to the ISG model, there were (1) significant individual differences in linear trait change across time as well as (2) a significant mean increase in trait mood over the course of the study. The latter findings are in line with a study by Csikszentmihalyi and Hunter (2003) who found a similar effect of week day on mood.

## Discussion

Researchers often analyze the longitudinal dynamics of psychological constructs via latent variable statistical models. LST models are most useful when the longitudinal course of a construct is characterized by a short-term state-variability process, that is, when individuals' true scores show situation-specific fluctuations, but do not involve changes of latent trait scores across time. In contrast, multiple-indicator LGC models are frequently used when researchers hypothesize that changes occurred in individuals' latent trait scores in addition to state-variability processes. The use of multiple-indicator LGC models for modeling trait changes over time has been recommended to overcome limitations of single-indicator LGC models (e.g., Geiser et al., 2013). In this article, we demonstrated that both LST and multiple-indicator LGC models can be specified not only within the SL-SEM, but also within the ML-SEM framework. In our discussion, we focus on the advantages and limitations of the ML-SEM parameterizations of these models.

### Advantages

Specifying LST and multiple-indicator LGC models as ML-SEM models leads to more compact model specifications compared to the corresponding SL-SEM parameterizations of the same models. This means that researchers can specify these complex longitudinal SEM models with fewer lines of code and also obtain a more compact output of parameter estimates for these models. On the practical side, this may lead to fewer errors in the model specification and a more straightforward processing and evaluation of the parameter estimates by the researcher.

For both LST and LGC models, the SL-SEM specification becomes inefficient when there are many waves of data, because a separate measurement model with time-specific latent state residual factors (ζ_*t*_) has to be set up for each time point (in the present example, we would have needed to include 56 individual state residual factors). In contrast, the ML-SEM specification remains the same, regardless of the number of time points involved. The only factor that leads to an increase in the model size in the ML-SEM specification is the number of indicators included in the analysis.

The ML-SEM approach allows researchers to fit LST and multiple-indicator LGC models in data situations in which the SL-SEM specification becomes very complicated (and sometimes virtually infeasible) due to a large number of observations per individual, individually-varying times of observations, unequally-spaced time points, or missing data. Although individually-varying and unequally-spaced times of observations pose less of a problem for LST models, these conditions create substantial challenges in the specification of LGC models, which depend heavily on the proper specification of the time metric (e.g., Mehta and West, 2000). The ML-SEM approach handles these conditions with ease through the inclusion of a Level-1 time variable, leading to a much more flexible specification than in the SL-SEM approach.

Another advantage of the ML-SEM approach is that it easily handles missing data due to drop-out of the study over time that satisfies the MAR condition. No imputation or specific missing data algorithms are required, as cases with longitudinal missing data are automatically included in the parameter estimation as long as they provide data for at least one measurement occasion^4^.

### Limitations

The ML-SEM approach makes implicit assumptions about the time-invariance of a number of parameters. Specifically, it is implicitly assumed that all intercepts, factor loadings, measurement error variances, and state residual factor variances are time-invariant, that is, take on the same values across time. This implies the assumption of strict measurement equivalence across time (Widaman and Reise, 1997) plus the assumption that the latent state residual variances remain constant at all time points. For LST models, this implies that the *Con*, *Ospe*, and *Rel* coefficients are time-invariant.

These assumptions are not completely unreasonable in practice, and they lead to compact and parsimonious models. In fact, many past applications of LST models within the SL-SEM framework have imposed similar constraints. On the other hand, these assumptions are clearly strong and may be violated in empirical applications. Therefore, it is typically recommended that researchers test them empirically, as violations of these assumptions may lead to bias in estimated parameters or confusion about the type of longitudinal process under study (Geiser et al., submitted). Testing these assumptions, however, is only possible within the SL-SEM, but not the ML-SEM specification of LST and LGC models.

Second, and related to the first issue, the ML-SEM specification in general yields different degrees of freedoms (*df*) and overall fit measures compared to the SL-SEM specification. The reason for this is that certain aspects of the models that increase the model *df* in the SL-SEM framework do not lead to an increase in the *df* in the ML-SEM framework. For example, the SL-SEM framework provides greater *df* for MTMS models with more indicators *or* more time points. In contrast, the *df* for the MTMS model in the ML-SEM framework can only be increased by increasing the number of indicators, but not by increasing the number of measurement occasions.

This issue is related to the first issue discussed above, namely the fact that the ML-SEM specification involves a number of implicit constraints with regard to the time-invariance of latent parameters. As we noted above, the assumption of time-invariant parameters is implicitly made, but is not testable in this framework. Therefore, ML-SEM versions of LST and LGC models will generally yield fewer *df* than their SL-SEM counterparts.

Given that the same longitudinal models will generally show fewer *df* in the ML-SEM as compared to the SL-SEM framework, their global fit (e.g., in terms of the chi-square test of model fit and related fit statistics) will tend to “look” better in the ML-SEM as compared to the SL-SEM framework, because some aspects of the models remain untested in the ML-SEM framework. In this regard, it is worth noting that also the independence model, which is used, for example, to calculate incremental fit statistics like the CFI, is also different in the ML-SEM compared to the SL-SEM framework.

The differences in *df* and model fit may confuse researchers and may also lead to problems in practice with regard to testing assumptions of longitudinal models. It should be noted, however, that this problem is not “new”—it simply does not seem to have received much explicit attention in the literature or appreciation among researchers. For example, as we pointed out earlier in this paper, it is well-known that single-indicator LGC models can be specified using either the ML or SL-SEM frameworks. In the specification of single-indicator LGC models as ML models, similar issues as demonstrated here for LST and multiple-indicator LGC models occur: In the SL-SEM framework, these models are typically overidentified with *df* > 0 and yield an overall chi-square test of model fit. In contrast, in the ML regression framework, no chi-square test of global model fit is available, despite the fact that the LGC models in the ML framework often include *more* restrictions than their SL-SEM counterparts such as, for example, equality constraints on the Level-1 error variances *Var*(ε).

That is, although it is implicitly assumed in the ML specification of single-indicator LGC models that the error variances are constant across time, this assumption is not subject to a global model fit test in the ML framework. In contrast, single-indicator LGC models are usually specified with freely estimated error variances in the SEM framework, and testing the assumption of equal error variances typically involves an explicit model fit comparison. In summary, researchers need to carefully consider whether the benefits of a simplified specification within the ML framework outweigh the costs of not being able to test certain underlying constraints that would be testable within the SL-SEM approach.

A third limitation of the ML-SEM approach is that the specification of multiple-indicator LGC models within the multilevel SEM framework involves the specification of random slope coefficients. For random slopes models, no global chi-square test of model fit is currently available in the software Mplus. Therefore, the fit of the multiple-indicator LGC models cannot be tested in the same way as in the SL-SEM framework. This is different for the LST model, which does not involve random slopes, and thus yields an overall chi-square fit test also in the ML-SEM specification (although the restrictions with regard to *df* etc. as discussed in the previous paragraph apply).

Model fit tests for multiple-indicator LGC models are routinely available in the SL-SEM specification. Once again, researchers have to carefully consider whether (1) the SL-SEM specification is feasible and (2) if not, whether the benefits of fitting the multiple-indicator LGC model in the ML-SEM specification outweigh the costs of not being able to test the model fit with a global fit statistic. Furthermore, future studies should examine the potential consequences of making incorrect (but untestable) assumptions in the ML-SEM framework for parameter estimate bias. This could be done by directly comparing the SL- and ML-SEM versions of the models with either actual or simulated data.

It is well-known from the time-series literature (e.g., Box and Jenkins, 1970) that autoregressive processes are common in longitudinal data with closely adjacent measurement occasions. A final limitation of the ML-SEM approach is that it does not provide an easy way to model more complex error or state residual structures, such as correlated residuals across time or autoregressive processes among latent state residuals (Cole et al., 2005). Especially in EMA studies, the presence of autoregressive processes must be expected, because time points are often closely adjacent. Complex residual covariance structures and autoregressive processes are more easily specified and tested in the SL-SEM approach. As an alternative to the ML-SEM approach, researchers dealing with individually-varying time points can specify longitudinal models with so-called TSCORES in Mplus, which allow including information on individually-varying times of observations (for an example, see Eid et al., 2012). Missing data can be handled within the SL-SEM framework by means of FIML estimation or MI.

Despite the above-mentioned limitations of the ML-SEM approach to modeling variability and trait-change processes, there are situations in which the SL-SEM specification is simply not feasible because of data characteristics like the ones described above. In these cases, the ML-SEM approach represents a viable alternative. In addition, given that the ML-SEM versions of LST and multiple-indicator LGC models are more easily specified than the corresponding SL-SEM models in many cases, the ML-SEM approach can be useful in a first analysis step in which the key characteristics of a longitudinal data set are explored before more complicated models are fit to the data.

### Relationship between LST models and multitrait-multimethod models for interchangeable raters

Researchers familiar with the literature on modern methods of multitrait-multimethod (MTMM) analysis may have wondered about similarities between the structure of LST and CFA-MTMM models. In fact, it can be shown that LST models like the ones presented here are very similar in terms of the underlying psychometric theory and model structure compared to MTMM models for interchangeable (randomly selected) methods. CFA-MTMM models for interchangeable methods (e.g., randomly selected employees rating supervisors) have recently been presented by Eid et al. (2008) as well as Koch et al. (submitted). Eid et al. (2008) and Koch et al. (submitted) showed that CFA-MTMM models for interchangeable methods can also be specified as ML-SEM models. These models have the same structure as the LST models presented here with the situations replaced by interchangeable methods (e.g., raters nested within targets). The same advantages and limitations of the ML-SEM approach apply in the MTMM case as well: The ML-SEM specification is convenient when there are many raters per target and/or when there is an unequal number of raters for each target.

Furthermore, the ML-SEM approach makes the implicit assumption that certain model parameters are invariant across raters (i.e., the method factor loadings, method factor variances, and measurement error variances). In the single-trait multi-method case, the *df* of the model can only be increased by adding more indicators, but not by adding more raters (methods). If researchers want to test the above equality assumptions, they have to specify the corresponding SL-SEM versions of these models if possible (see Nussbeck et al., 2009, for an example).

## Conclusion

LST and LGC models are frequently applied to analyze longitudinal data in psychology and the social sciences. The specification of these models within the ML-SEM framework can be practical under certain circumstances, but also comes with certain limitations. The purpose of this article was to inform researchers about the potential benefits and limitations of employing a ML-SEM approach to estimating the parameters of LST and multiple-indicator LGC models in complex longitudinal designs.

## Author note

This research was funded by a grant from the National Institutes on Drug Abuse (NIH-NIDA), grant #1 R01 DA034770-01 awarded to Ginger Lockhart and Christian Geiser. The data used in the empirical application was collected under NIH Research Grant U01 HL97839 to Kim Reynolds, Principal Investigator, Claremont Graduate University, through the Obesity Related Behavioral Intervention Trials (ORBIT) initiative funded by the National Heart, Lung, and Blood Institute and the National Institute of Child Health and Human Development.

### Conflict of interest statement

The authors declare that the research was conducted in the absence of any commercial or financial relationships that could be construed as a potential conflict of interest.

## Footnotes

^1^In the present article, for the sake of simplicity, we ignore potential further levels such as, for example, individual children nested within school class rooms, but note that additional levels could in principle be included in all models presented here.

^2^In all path diagrams in this paper, paths without an explicit coefficient indicate paths that are fixed to 1; paths that are set to zero, including zero intercepts, are not explicitly shown in the figures.

^3^Note that McArdle's (1988) SGM could be specified in a similar way in the ML-SEM framework. The only difference between the GSG and the SGM specification is that in the SGM, the trait factor loadings (λ_*i*_) have to be set equal to the corresponding latent state residual factor loadings (γ_*i*_) for all indicators (i.e., λ_*i*_ = γ_*i*_ for all *i*).

^4^Note that missing data on individual observed variables (e.g., a person X provides data at time *t*; however, the person only provides responses to Items 1 and 2, but not Item 3) would still require additional missing data handling techniques even in the ML-SEM approach, and that the assumption of MAR is not testable in general.

## Appendix

### Mplus scripts for the specification of the STMS, MTMS, GSG, and ISG models as multilevel structural equation models

#### STMS model

title: Singletrait-multistate model
       (multilevel SEM specification)

        This model is shown in Figure 1C

data: file = subam.dat; ! Name of data file

variable: names = ID Time Y1 Y2 Y3;
          ! variable names

          missing = ALL (-9999 -1);
          ! missing value code

          usevar = ID Y1 Y2 Y3 Time;
          ! variables used in the analysis

          cluster = ID; ! Cluster variable
          defining Level-2 units

          within = time; ! Specification of
          time as a Level-1 variable

analysis: type = twolevel; ! Multilevel
          analysis with two levels

          estimator = ML; ! Maximum
          likelihood estimation
model:

! Level-1 part of the model

%within%

! Latent state residual variable

zeta by Y1@1 Y2 Y3;

! Regression of indicators on time variable

! Note that the effect of time is fixed
  to zero in this model

Y1 Y2 Y3 on time@0;

! Estimate error variances Var(epsilon_i)

Y1-Y3^*^;

! Level-2 part of the model

%between%

! Item-Specific Trait Factors

ksi1 by Y1@1;

ksi2 by Y2@1;

ksi3 by Y3@1;

! Common Trait factor

ksi by ksi1@1 ksi2 ksi3;

! Estimate means and intercepts

[ksi1@0 Y1-Y3@0]; ! Set intercepts to zero

[ksi^*^]; ! Common trait factor mean

[ksi2^*^]; ! Intercept alpha_2

[ksi3^*^]; ! Intercept alpha_3

! Fixing Level-2 residual variances to zero

Y1-Y3@0; ksi1-ksi3@0;

output: sampstat stdyx; ! Requesting sample
                          statistics and
                          completely

                          ! standardized
                          parameter estimates

#### MTMS model

title: multitrait-multistate model
       (multilevel SEM specification)

      This model is shown in Figure 1D

data: file = subam.dat; ! Name of data file

variable: names = ID Time Y1 Y2 Y3;
          ! variable names

          missing = ALL (-9999 -1);
          ! missing value code

          usevar = ID Y1 Y2 Y3 Time;
          ! variables used in the analysis

          cluster = ID; ! Cluster variable
          defining Level-2 units

          within = time; ! Specification of
          time as a Level-1 variable

analysis: type = twolevel; ! Multilevel
          analysis with two levels

          estimator = ML; ! Maximum
          likelihood estimation
model:

! Level-1 part of the model

%within%

! Latent state residual variable

zeta by Y1@1 Y2 Y3;

! Regression of indicators on time variable

! Note that the effect of time is fixed to
zero in this model

Y1 Y2 Y3 on time@0;

! Estimate error variances Var(epsilon_i)

Y1-Y3^*^;

! Level-2 part of the model

%between%

! Item-Specific Trait Factors

ksi1 by Y1@1;

ksi2 by Y2@1;

ksi3 by Y3@1;

! Estimate latent trait means

[Y1-Y3@0]; ! Set intercepts to zero

[ksi1-ksi3^*^]; ! Estimate trait factor means

! Estimate trait factor covariances

ksi1-ksi3 with ksi1-ksi3^*^;

! Fixing Level-2 residual variances to zero

Y1-Y3@0;

output: sampstat stdyx; ! Requesting sample
                          statistics and
                          completely
                          ! standardized
                          parameter estimates

#### GSG model

title: generalized second-order growth model
       (multilevel SEM specification)

       This model is shown in Figure 4C

data: file = subam.dat; ! Name of data file

variable: names = ID Time Y1 Y2 Y3;
          ! variable names

          missing = ALL (-9999 -1);
          ! missing value code

          usevar = ID Y1 Y2 Y3 Time;
          ! variables used in the analysis

          cluster = ID; ! Cluster variable
          defining Level-2 units

          within = time; ! Specification of
          time as a Level-1 variable

analysis: type = twolevel; ! Multilevel
          analysis with two levels

          estimator = ML; ! Maximum
          likelihood estimation
model:

! Level-1 part of the model

%within%

! Latent state residual variable

zeta by Y1@1 Y2 Y3;

! Regression of indicators on time variable
with random slopes

! The effect of time is linear

ksi_lin1 | Y1 on time;

ksi_lin2 | Y2 on time;

ksi_lin3 | Y3 on time;

! Estimate error variances Var(epsilon_i)

Y1-Y3^*^;

! Level-2 part of the model

%between%

! Item-Specific Intercept Factors

ksi_int1 by Y1@1;

ksi_int2 by Y2@1;

ksi_int3 by Y3@1;

! Common intercept factor

ksi_int by ksi_int1@1

           ksi_int2 (lambda2)

           ksi_int3 (lambda3);

! Common linear slope factor

ksi_lin by ksi_lin1@1

           ksi_lin2 (lambda2)

           ksi_lin3 (lambda3);

! Estimate latent intercept and slope
  factor means

[Y1-Y3@0 ksi_int1@0 ksi_lin1@0];
! Set intercepts to zero for indicators and

! reference variables

[ksi_int2^*^]; ! Estimate intercepts

[ksi_int3^*^];

[ksi_int^*^ ksi_lin^*^]; ! Estimate growth
                         factor means

! Estimate common growth factor covariance

ksi_int with ksi_lin^*^;

! Fixing Level-2 residual variances
  to zero

Y1-Y3@0; ksi_int1-ksi_int3@0;
ksi_lin1-ksi_lin3@0;

output: sampstat stdyx; ! Requesting sample
                          statistics and
                          completely
                          ! standardized
                          parameter estimates

#### ISG model

title: indicator-specific growth model
       (multilevel SEM specification)

        This model is shown in Figure 4D

data: file = subam.dat; ! Name of data file

variable: names = ID Time Y1 Y2 Y3;
          ! variable names

          missing = ALL (-9999 -1);
          ! missing value code

          usevar = ID Y1 Y2 Y3 Time;
          ! variables used in the analysis

          cluster = ID; ! Cluster variable
          defining Level-2 units

          within = time; ! Specification of
          time as a Level-1 variable

analysis: type = twolevel; ! Multilevel
          analysis with two levels

          estimator = ML; ! Maximum
          likelihood estimation
model:

! Level-1 part of the model

%within%

! Latent state residual variable

zeta by Y1@1 Y2 Y3;

! Regression of indicators on time variable
  with random slopes

! The effect of time is linear

ksi_lin1 | Y1 on time;

ksi_lin2 | Y2 on time;

ksi_lin3 | Y3 on time;

! Estimate error variances Var(epsilon_i)

Y1-Y3^*^;

! Level-2 part of the model

%between%

! Item-Specific Intercept Factors

ksi_int1 by Y1@1;

ksi_int2 by Y2@1;

ksi_int3 by Y3@1;

! Estimate latent intercept and slope
  factor means

[Y1-Y3@0]; ! Set intercepts to zero

[ksi_int1-ksi_int3^*^ ksi_lin1-ksi_lin3^*^];

! Estimate growth factor means

! Estimate growth factor covariances

ksi_int1-ksi_int3 ksi_lin1-ksi_lin3
with ksi_int1-ksi_int3^*^ ksi_lin1-
ksi_lin3^*^;

! Fixing Level-2 residual variances to zero

Y1-Y3@0;

output: sampstat stdyx; ! Requesting sample
                          statistics and
                          completely

                      ! standardized
                        parameter estimates
